# A mechanistic computational model of HGF-VEGF-mediated endothelial cell proliferation and vascular permeability

**DOI:** 10.1016/j.isci.2025.113199

**Published:** 2025-07-24

**Authors:** Rebeca Hannah de Melo Oliveira, Akash Patil, Brian H. Annex, Arvind P. Pathak, Aleksander S. Popel

**Affiliations:** 1 Department of Biomedical Engineering, School of Medicine, Johns Hopkins University, Baltimore, MD 21205, USA; 2 Medical College of Georgia at Augusta University, Augusta, GA, USA; 3 Department of Radiology and Radiological Science, School of Medicine, Johns Hopkins University School of Medicine, Baltimore, MD, USA; 4 Sidney Kimmel Comprehensive Cancer Center, Baltimore, MD, USA

**Keywords:** Integrative aspects of cell biology, Experimental models in systems biology, *In silico* biology, Biological constraints

## Abstract

Hepatocyte growth factor (HGF) and vascular endothelial growth factor (VEGF) are important pro-angiogenic factors in angiogenesis-dependent diseases. While sharing some signaling pathways, their contrasting effect on vascular permeability remains under investigation. To explore how these factors promote angiogenesis, we developed, calibrated, and validated a data-driven mechanistic computational model of HGF and VEGF signaling in endothelial cells (ECs). We proposed that variations in permeability profiles may stem from RAC1-PAK1 activation via site-specific phosphorylation. By introducing permeability and proliferation indices, our simulations indicated a dose-dependent effect of VEGF that hampered the ability of HGF to promote vascular stability. Our simulations indicate that HGF did not require VEGFR2 activation to affect permeability and proliferation. This model has the potential to be applicable and helpful in analyzing angiogenesis-dependent diseases. It provided insights into the mechanisms of EC proliferation and vascular permeability induced by HGF and VEGF and permitted evaluation of their separate or combined effects.

## Highlights

• Integrative mechanistic computational model of HGF/VEGF signaling in endothelial cells
• Dose-based modeling revealed that HGF can counteract VEGF-induced EC permeability
• Sensitivity analysis revealed context-specific regulators of EC permeability
• PAK1 phosphorylation sites drove VEGF/HGF-specific differences in EC permeability

## Graphical abstract

**Figure:**
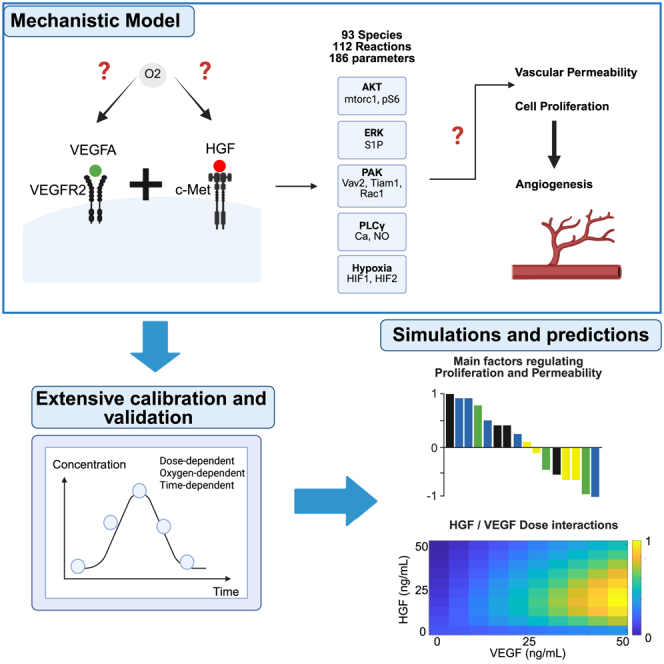

## Introduction

Angiogenesis, the formation of new blood vessels from pre-existing ones, is a highly complex process regulated by different cells and environmental factors. Among the main studied angiogenesis-dependent diseases are cancer, ocular diseases (such as neovascular age-related macular degeneration and diabetic retinopathy [DR]), and diseases marked by ischemia, such as atherosclerosis^1^ and peripheral arterial disease (PAD). DR affects more than 100 million people worldwide and is a leading cause of blindness.^2^ PAD affects more than 250 million people globally and, as of today, has no known cure.^3^^,^^4^^,^^5^ Anti- and pro-angiogenesis therapies have been explored as treatments, including regulating growth factors and their effects on different signaling pathways. The vascular endothelial growth factor (VEGF) and the hepatocyte growth factor (HGF) are two important pathways regulating angiogenesis that have been studied over the years as potential therapeutic targets for angiogenesis-related diseases. These pathways have a complex relationship and can influence vascular permeability and endothelial cell (EC) proliferation. Therefore, a mechanistic understanding of their interaction and distinct signaling effects as separate or combined therapies can help understand and improve their use for regulating angiogenesis and treating angiogenesis-related conditions. Computational models provide the flexibility necessary to investigate, understand, and evaluate the mechanistic effects of this therapeutic approach.

**Figure 1:**
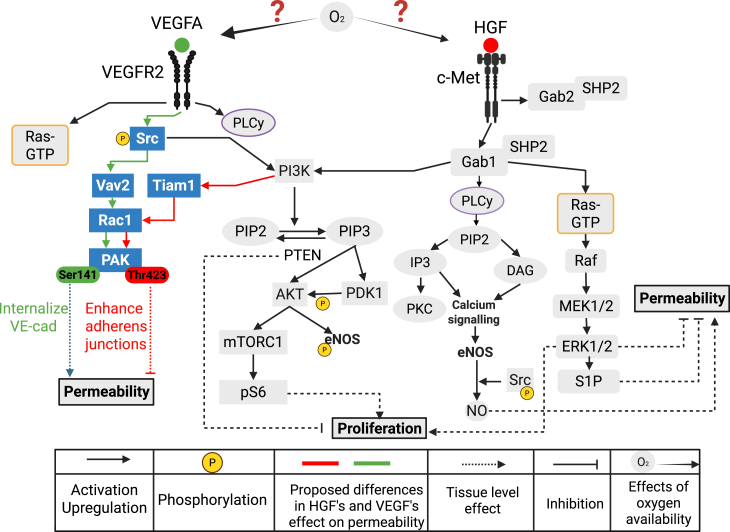
Model diagram including VEGF and HGF signaling to regulate vascular permeability and cell proliferation HGF and VEGF activate their receptors, cMet and VEGFR2, inducing downstream activation of PI3K, Akt, and ERK. Downstream of cMet activation, Gab2 competes with Gab1 to bind with phosphorylated cMet. Through PI3K activation, HGF can lead to PAK activation to enhance adherens junctions, limiting permeability. Meanwhile, VEGF can lead to differential activation of PAK, leading to vascular endothelial cadherins (VE-cad) internalization, which increases vascular permeability. Potential PAK residues stimulated by VEGF or HGF are Ser141 and Thr423. Nitric oxide (NO) is downstream of both pathways and influences vascular permeability. Both factors induce cell proliferation through PI3K and ERK activation, while PTEN exerts an opposite effect, limiting proliferation. Dashed lines represent tissue-level effects, and solid lines represent pathway signaling. For clarity, Ras-GTP and PLCγ are shown as simplified boxed nodes downstream of VEGFR2; their corresponding pathways are depicted in full detail on the HGF/c-Met side. The border colors of these boxes indicate the associated signaling branch. Potential mechanisms through which VEGF and HGF establish different effects are shown in green (VEGF related) and red (HGF related). The influence of different oxygen levels on VEGF and HGF signaling is also investigated.

VEGF and HGF act through their respective receptors—VEGFRs for VEGF and cMet for HGF—to regulate events that influence angiogenesis. Many factors regulating vascular permeability and EC proliferation lie downstream of the VEGF and HGF pathways and could be common to both. For example, both stimuli can affect Akt and ERK phosphorylation and calcium and nitric oxide (NO) signaling^6^^,^^7^^,^^8^^,^^9^^,^^10^ (Figure 1). Additionally, hypoxia regulates several steps of HGF and VEGF signaling, including receptor expression,^11^^,^^12^ calcium dynamics,^13^ NO dynamics,^14^ and growth factor secretion.^15^^,^^16^^,^^17^^,^^18^ EC proliferation is affected by many stimuli, as well as pathological conditions.^19^ Studies with VEGF stimulation of angiogenesis have shown that VEGF promotes cellular proliferation and affects vascular stability, leading to leaky vasculature.^20^ HGF, while promoting cellular proliferation, stabilizes the vasculature and limits tissue inflammation and necrosis.^21^^,^^22^ Although VEGF is recognized for its pro-angiogenic activity in tumors and retinopathy,^23^^,^^24^^,^^25^^,^^26^^,^^27^^,^^28^ clinical trials testing its application in treating ischemic diseases such as PAD have been repeatedly unsuccessful.^5^^,^^29^ VEGF therapy promotes the formation of new blood vessels that are characterized by their hyperpermeability, which can lead to unstable vasculature and leakiness, compromising tissue reperfusion.^30^ HGF is also recognized for its pro-angiogenic activity, but while VEGF promotes angiogenesis in an NO-dependent manner, stimulating permeability and inflammation, HGF has anti-inflammatory effects and reduces vascular permeability.^31^

Therapeutic interventions have focused on improving blood circulation through pro-angiogenesis therapies, and clinical trials have shown promising results in treating PAD with HGF.^32^^,^^33^ Recent studies that have combined HGF with VEGF suggest that HGF may require enhanced VEGF signaling to exert its angiogenic effects. Although VEGF and HGF have several points of interconnection throughout their downstream signaling and even through receptor regulation,^7^^,^^21^^,^^34^ their mechanism of action for controlling EC permeability and proliferation can differ, leading to different outcomes in improving ischemia recovery. Studying the mechanistic interactions between HGF and VEGF can help us understand their complex interactions and the potential interdependence of their effects. Moreover, such interactions can help us determine targets and potential combinations that optimize the therapeutic effects of these factors under different disease conditions.

Due to the large number of interactions in signaling pathways related to EC permeability and proliferation after stimulation by VEGF and/or HGF, as represented in Figure 1, the system’s complexity might preclude experimental evaluation of the mechanisms involved. In the past, computational biology models have helped us understand and study systems as intricate as this.^35^ Specifically, our group designed models to evaluate the dynamics of VEGF signaling in EC through different VEGF receptors, Akt and ERK activation, NO and Ca^2+^ signaling, Dll4-Notch1, and Ang-Tie dynamics and hypoxia sensing through the HIF pathway.^9^^,^^10^^,^^36^^,^^37^^,^^38^^,^^39^^,^^40^^,^^41^^,^^42^^,^^43^

Other researchers have also successfully employed mechanistic computational models of signaling pathways to enlighten and comprehend various mechanisms involved in angiogenesis in disease or healthy scenarios.^44^^,^^45^^,^^46^^,^^47^^,^^48^ Since no previous model has captured the complex VEGF-HGF crosstalk in detail, this is the main motivation and contribution of the present work. Using experimentally informed models allows us to propose and test hypotheses to understand highly complex biological mechanisms that regulate EC behavior. Based on a solid methodological formulation and insights from past models, we propose investigating the VEGF and HGF signaling mechanisms that regulate EC permeability and proliferation. We aim to answer four main questions regarding the HGF-VEGF relationship in the context of pro-angiogenesis effects: (1) which mechanisms might mediate the divergent effects of HGF and VEGF on vascular permeability? (2) How does early hypoxia (<2 h) interfere with signaling through either ligand? (3) Does HGF depend on VEGF activation of VEGFR2 to exert its effect? Moreover, (4) how can we combine VEGF and HGF to induce EC proliferation while maintaining vascular stability?

To answer these questions, we designed and present a data-driven mechanistic computational model of HGF and VEGF in ECs to evaluate how HGF and VEGF differentially and synergistically affect vascular permeability and EC proliferation and promote angiogenesis under different oxygen conditions (i.e., normoxia or hypoxia). We designed our model based on good modeling practices, including structural and practical identifiability analysis (PIA), parameter optimization, and uncertainty quantification. Using global sensitivity analysis of the optimized model, we find the set of parameters in each pathway with a higher influence on determining EC proliferation and vascular permeability. Additionally, we simulate time and dose responses to VEGF and HGF singly or as combined therapy, predicting their effect on modulating important aspects of angiogenesis under normal or deprived oxygen conditions. The model we present can be applied to understanding the microvascular events that occur when HGF and VEGF are given singly or as combined therapy and can be used to investigate potential pro-angiogenesis therapeutic interventions.

## Results

### A mechanistically calibrated computational model recapitulates experimental results

**Figure 2:**
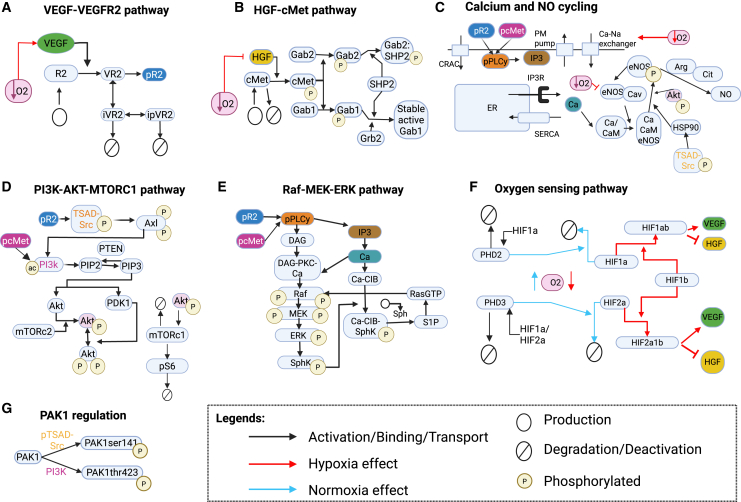
Detailed diagram of pathways included in the model (A) VEGFR2 phosphorylation induced by VEGF, receptor internalization, degradation, and recycling. (B) HGF activates c-Met, leading to Gab1 and Gab2 phosphorylation and activation. (C) VEGFR2 and c-Met phosphorylation lead to IP3 production, which binds to its receptor and allows calcium release into the cytoplasm from the endoplasmic reticulum (ER), increasing intracellular calcium concentration. Calcium activates eNOS, causing NO production, a process also regulated by Akt, HSP90, and arginine (Arg). (D) Phosphorylated cMet activates PI3K, and phosphorylated VEGFR2 leads to Src phosphorylation and PI3K activation, causing downstream phosphorylation of Akt mediated by mTORC2. (E) Phosphorylated receptors lead to PLCγ phosphorylation and activation, which causes ERK phosphorylation and IP3-mediated increase in intracellular calcium. (F) HIF1/2α is degraded under normoxia but stabilized under hypoxia. Stable HIFs dimerize with HIF1b, regulating VEGF and HGF. (G) Through activation of Src and PI3K, PAK1 can be phosphorylated at different residues, influencing cell response differently. To facilitate interpretation of crosstalk between pathways, shared or “linking” proteins (e.g., pR2, pcMet, PI3K, pAKT, and pPLCγ) are highlighted with distinctive colors, enabling tracking of their roles across signaling modules. VEGF and HGF are indicated in green and yellow, respectively. Basal production and degradation rates are modeled for selected key species.

The computational model we present includes detailed reactions and mechanisms, further detailed in the STAR Methods. Figure 2 illustrates the pathways and reactions included in the model.

**Figure 3:**
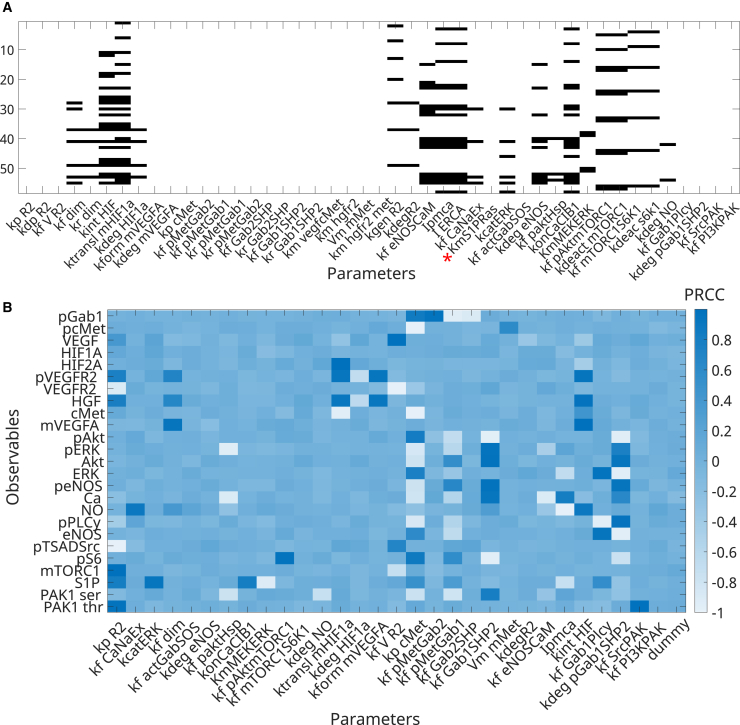
Identifiability analyses identify unidentifiable unknown parameters (A) Identifiability tableau for unknown parameters are generated through GenSSI 2.0. Empty columns represent structurally unidentifiable parameters. The red asterisk marks unidentifiable parameters found with STRIKE-GOLDD2.0. (B) Sensitivity analysis of observables to SI unknown parameters after the exclusion of collinearity, using PRCC. A dummy parameter was included in the analysis, indicating a lack of sensitivity. Parameters that show PRCC ≥0.1 were considered practically identifiable.

The final integrative model comprises 93 species, 186 parameters, 112 reactions, and 93 ODEs (with the variable jcrac being explained by an ODE along with the 93 species and HIF1 β being considered constant). Of the initial 186 parameters, after searching the literature, 45 values were unknown, and 141 were obtained from previous data in the literature. Next, we performed structural and practical identifiability analyses, according to the methodology presented in the STAR Methods. From the initial 45 unknown parameters, we found a subset of 21 structurally identifiable parameters through GenSSI and 44 through STRIKE-GOLDD. Km_S1PRas was the only unknown parameter found as unidentifiable by both toolboxes, and therefore, its value was assumed and fixed as presented in Table S2. The structural identifiability analysis (SIA) results obtained with GenSSI and STRIKE-GOLDD are presented in Figure 3A (the identifiability tableau generated by GenSSI and the asterisk marking the STRIKE-GOLDD result). Next, from the collinearity evaluation, we found a total of 30 structurally identifiable non-collinear unknown parameters. These are listed in Table S3 and marked as evaluated.

From the partial rank correlation coefficient (PRCC) analysis, out of the 30 parameters that were structurally and practically identifiable, we identified 26 unknown parameters to be influential (PRCC > 0.1 for at least one observable). These were selected for model calibration. Figures 3A and 3B summarize the SIA and PIA results, respectively. Complete lists are provided in Tables S2 and S3. In Figure 3B, colors indicate stronger or weaker influence on each observable. Observables are listed on the *y* axis of the figure, and evaluated parameters are on the *x* axis. Parameters that showed higher positive or negative influence on at least one of the observables were selected for fitting.

**Figure 4:**
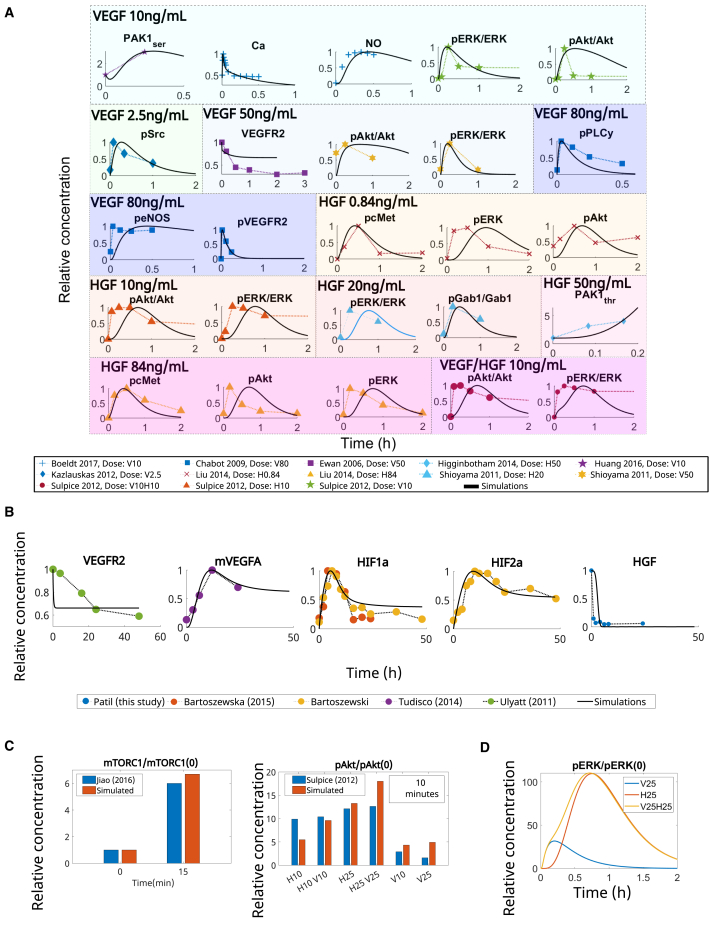
Model calibration and validation reproduces literature data (A) Simulated time response under normoxic conditions of species included in the model compared to data reported by other groups. VEGF and HGF dosage (ng/mL) are listed in the legend, according to the dose reported in the referenced experiment. (B) Simulated time response under hypoxia (1% O_2_), with no additional dose of VEGF/HGF. Basal values of VEGF and HGF are set to the representative amount of 1e−5 μM, to observe the effects of hypoxia on VEGF and HGF concentration over time. Patil (this study) refers to measurements performed by our group, as detailed in the methodology for performing ELISA. In (A) and (B), values are normalized to their maximum concentration or initial concentration (PAK1_ser_ and PAK1_thr_). (C) Dose response of mTORC1 and pAkt relative to their initial concentration. (D) Time response of pERK relative to its initial concentration. Abbreviations are as follows: H, HGF; V, VEGFA. Numbers represent the dose in ng/mL (e.g., V25H25 is VEGF at 25 ng/mL and HGF at 25 ng/mL). Color blocks separate based on stimulus, as annotated on the left upper corner of each color block.

From model calibration, we found that our model recapitulated the experimental data. Our post-fitting results compared to experimental data points are presented in Figure 4. The values found for each parameter are listed in Table S10.

Figure 4A shows our post-calibration simulation results (solid lines) compared with experimental data reported in the literature for normoxia conditions, with VEGF and HGF stimulation.^34^^,^^49^^,^^50^^,^^51^^,^^52^^,^^53^^,^^54^^,^^55^^,^^56^ The respective doses used are listed in Figure 4A. Figure 4B compares the simulated responses under hypoxia (1% O_2_) conditions with experimental data reported in the literature^11^^,^^57^^,^^58^^,^^59^ or collected by us (for HGF behavior). The methodology employed for collecting the data for HGF is presented in the STAR Methods, and the raw results are included as supplemental information (Tables S8 and S9; Figures S2 and S3).

As a validation step, we compared the dose responses of our model to data reported in the literature. Figure 4C presents the dose response of pAkt and mTORC1 relative to their initial concentrations under normoxia conditions, given stimulation with VEGF or HGF, compared to reported data found in the literature.^34^^,^^60^ Finally, Figure 4D presents the simulated results of pERK concentration relative to its initial concentration under normoxia conditions after stimulation of the network with 25 ng/mL of VEGF alone, HGF alone, or their combination.

Comparing experimental data to our model simulations, we found a good correspondence after calibration. Simulations under normoxia showed the time-dependent effect of VEGF and HGF on downstream species, with both stimulations upregulating pERK and pAkt. Under hypoxia, we observed the acute effect of oxygen deprivation on the expression of both VEGF and HGF. While hypoxia quickly increased the amount of VEGF, it depleted the amount of free HGF in less than 1 h (seen experimentally and reproduced by our simulations). Previous studies^22^^,^^34^ have reported increased ERK phosphorylation for combined VEGF/HGF stimulation than with either alone and increased ERK phosphorylation by stimulation with HGF than with VEGF. We tested our model’s ability to reproduce these findings, and, as shown in Figure 4D, our simulations agreed with them.

From the Runs test, our results showed all *h* values equal to zero, and the *p* values are presented in Table 1, with an average value (over all species) of *p* = 0.648. This corroborated the goodness of fit in our calibrated model. The lowest *p* value found was for HIF2a, which could be improved by acquiring more data for calibration.

**Table 1:** Runs test indicated goodness of fit on model optimization

Observable	*p*
pcMet	1.00
pGab1/Gab1	1.00
pAkt	0.33
pERK	0.38
pERK/ERK	0.81
pAkt/AKT	0.79
pPLCy	0.40
peNOS	0.40
NO	1.00
Ca	0.30
pSrc	0.67
PAK1thr	0.67
PAK1ser	1.00
HIF1a	0.83
HIF2a	0.05
HGF	0.67
mVEGFA	0.40
VEGFR2s (hypoxia)	1.00
VEGFR2s (normoxia)	0.27
pVEGFR2	1.00

**Figure 5:**
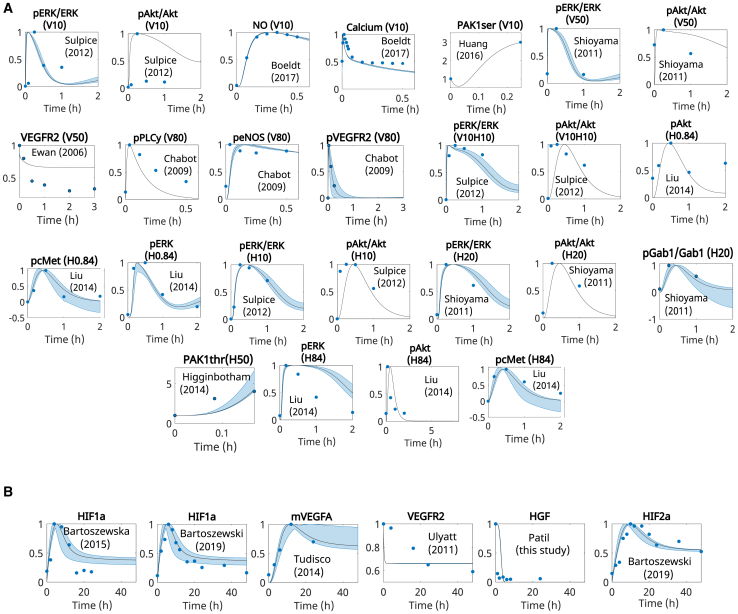
Model predictions closely follow experimental data (A) Confidence intervals for model fitting to (A) normoxic conditions with varying stimulation by VEGF (V) or HGF (H). (B) The equivalent stimulus used in each case is included in the title of the subplots, where the number refers to the dose in ng/mL and (B) hypoxia, with no initial stimulation. Circles represent experimental data collected from the literature cited within each subplot. HGF measurements under hypoxia were performed by our group, and are annotated as “Patil (this study).” The methodology employed for performing ELISA is described in the STAR Methods. Predicted data are represented as mean ± 95% confidence intervals.

Our uncertainty quantification analysis using 95% Bootstrap confidence intervals showed that the model calibration led to experimental results within the simulated confidence intervals, or close to them in most cases, under normoxia (Figure 5A) and hypoxia (Figure 5B) stimulations. With our validated model, we performed simulations to evaluate the effect of VEGF and HGF on cell proliferation and vascular permeability during early dynamics under normoxia and hypoxia (<2 h).

### Mono- or dual-stimulation by VEGF, HGF, and oxygen availability alters the effects of various model parameters on the PrI and PbI

To determine which mechanisms might mediate the divergent effects of HGF and VEGF on regulating vascular permeability, we first performed a thorough literature search for mechanisms downstream of VEGF or HGF that could lead to opposing effects on permeability regulation for different EC types. Since both factors upregulate the pro-permeability factor NO but have opposing effects on permeability, it is likely that an alternative route that is not downstream of NO upregulation is responsible for HGF limiting permeability. Our search indicated that signaling through Rac1 leading to PAK1 phosphorylation at different residues could be a mechanism regulating permeability. Previous work explored this mechanism in human umbilical vein EC (HUVEC) and pulmonary ECs, indicating that Rac1 interactors Tiam1, Asef, and Vav2 were potential modulators of PAK1 phosphorylation.^53^^,^^61^^,^^62^ HGF stimulates Tiam1 and Asef and confers protective effects on the endothelial barrier, while VEGF stimulates Vav2, causing an increase in permeability.^53^^,^^62^^,^^63^^,^^64^

Next, to evaluate the effects of hypoxia and differential stimulation by VEGF and HGF on cell proliferation (i.e., the PrI) and vascular permeability (i.e., the PbI), we analyzed their sensitivity to differential stimulation by the growth factors and under various oxygen conditions. Finally, to assess the HGF dependency of VEGF activation of VEGFR2 and how to combine VEGF and HGF to induce proliferation while maintaining vascular stability, we simulated the dose response of the PrI and PbI to various combinations of VEGF and HGF.

**Figure 6:**
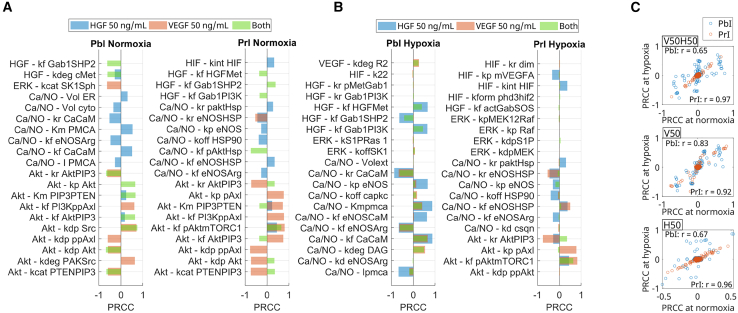
System stimulation and oxygen availability alter the effects of various model parameters on PrI and PbI (A) Top 10 positive and negative influencers of PbI and PrI given stimulation by HGF, VEGF, or both at 1 h. Parameters are ordered and annotated according to their signaling pathway group (HGF, VEGF, Akt, ERK, Ca/NO, and HIF) for clarity. (B) Significant influencers of PbI (left) or PrI (right) under hypoxia following VEGF and HGF stimulation. (C) Each point represents a model parameter, plotting its PRCC value relative to PbI (blue circles) or PrI (red circles) at 1 h under normoxia (*x* axis) against its corresponding PRCC value at 1 h under hypoxia (*y* axis). Thus, the number of points corresponds to the number of evaluated parameters. Correlation coefficients (r values) were calculated separately for PbI and PrI.

Figure 6A shows the top 10 parameters that exerted the greatest influence on PbI or PrI. We observed that, under HGF stimulation, parameters related to calcium and NO regulation were more potent influencers of the PbI, although the degradation of cMet and the binding of Akt to PIP3 also played a role in defining the PbI. Meanwhile, under VEGF stimulation, Akt-related parameters showed a greater influence on regulating the PbI. Combining VEGF and HGF showed that, while some of the influencers identified from single stimulation by VEGF or HGF remained, other reactions also contributed to the PbI, such as the interaction of Gab1 and SHP2.

When observing the influencers of the PrI under normoxia (Figure 6A), we found that parameters related to the mTORC1, NO, and HIF pathways were the most potent regulators under stimulation by HGF alone. Meanwhile, given VEGF stimulation alone, parameters within the Akt pathway prevailed among the top influencers of PrI. Under stimulation by both growth factors, parameters in the Akt and HGF pathways were top influencers of PrI, with Gab1 interaction with SHP2 being positively associated with PrI (which would indicate an increase in proliferation). In contrast, the interaction of HGF with cMet and Gab1 with phosphatidylinositol 3-kinase (PI3K) showed a negative association with the PrI.

We then evaluated which parameters mainly influenced PrI and PbI under hypoxia, given different combinations of HGF and VEGF, as indicated in Figure 6B. Our results suggested that the significant influencers of PbI varied more depending on oxygen availability than those of PrI. Specifically, comparing the results for top influencers of the PbI under normoxia (Figure 6A) with those under hypoxia (Figure 6B), the degradation of DAG (kdeg DAG), the binding of eNOS to calmodulin (kf eNOSCaM), the binding of Gab1 to PI3K (kf Gab1PI3K), the binding of HGF and cMet (kf HGFMet), and the phosphorylation of eNOS (kp eNOS) were among the top 10 leading influencers of PbI under hypoxia, which was not the case under normoxia.

Observing the major influencers of PrI under hypoxia and comparing their PRCC values given stimulation with HGF, VEGF, or their combination (Figure 6B), we noted that the combination of HGF and VEGF led to a change in the PRCC signal of unbinding of Akt and PIP3 (kr AktPIP3) and the phosphorylation of pAxl (kp pAxl). This change indicated that combinatory stimulation could lead to interactions that diminished or reversed the effect of these parameters on the PrI under hypoxia (e.g., kp pAxl and kr AktPIP3). Non-linear interactions between the pathways could also be a reason for such behavior.

Figure 6C shows scatterplots illustrating the correlation between the *x* and *y* axes under normoxia and hypoxia. We observed that the PRCCs of parameters on the PrI were linearly correlated under normoxia and hypoxia, regardless of the stimulation (r > 0.9). Meanwhile, we observed a weaker correlation (r = 0.65, for dual stimulation, r = 0.83 for VEGF stimulation, and r = 0.67 for HGF stimulation) on the PRCC of parameters relative to the PbI, with a stronger correlation seen in the case of stimulation by VEGF alone. The r values indicated a stronger positive correlation for PrI than PbI between the PRCCs under hypoxia vs. normoxia. This analysis suggests a higher influence of hypoxia on parameters that influence permeability than on those that regulate proliferation, agreeing with our previous results.

In summary, our global sensitivity analyses through PRCC under hypoxia and normoxia with various stimulations by VEGF and HGF reinforced the need to consider case-specific interventions, as alterations in oxygen levels and stimulation led to differences in the PRCC results. We found indications of potential non-linearities and complex relationships between reactions downstream of VEGF and HGF under normoxia and hypoxia, and our model suggested a higher impact of hypoxia on markers of permeability than on those of proliferation. Given our interest in the differential effect of VEGF and HGF on regulating permeability, our next step was to assess the direct and indirect influences of species included in the PbI equation given different system stimulations.

### PAK1_thr_ is a significant regulator of the acute PbI response

**Figure 7:**
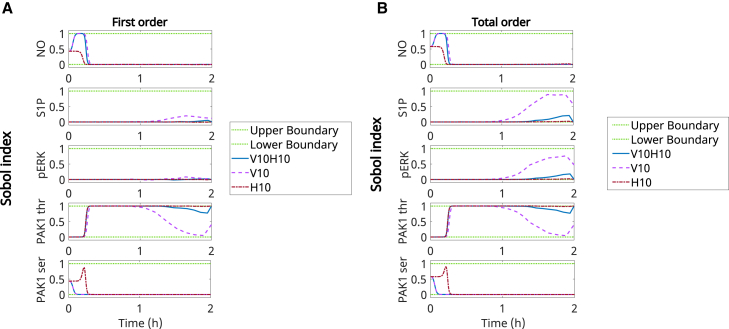
Temporal and stimulus-dependent sensitivity of PbI reveals PAK1thr as key regulator First-order (A) and total-order (B) Sobol sensitivity analysis indicated the temporal and dose-dependent influence of NO, S1P, pERK, PAK1ser, and PAK1thr on the PbI. Time variations on the Sobol index for each species under different initial stimulations by VEGF and/or HGF. H10 = HGF 10 ng/mL, V10 = VEGF 10 ng/mL, H10V10 = HGF and VEGF at 10 ng/mL during 2 h post stimulation.

Figure 7A shows the first-order responses given different initial stimuli and indicates that PAK1thr had a higher influence during most of the 2-h simulation, with NO and PAK1_ser_ presenting a higher effect at the initial moments. Given VEGF stimulation, PAK1_thr_ showed a transient effect that was reduced after 1 h of simulation. Stimulation with HGF alone or combined with VEGF led to a more durable impact of PAK1_thr_ on the PbI. Meanwhile, S1P and pERK presented a lower overall direct effect, increasing closer to the 2-h time point. In short, this analysis suggested that PAK1_thr_ was a key regulator of PbI during the 2-h simulation, with its dynamic effect depending on the system stimulation. Additionally, NO and PAK1_ser_ were more influential earlier during the simulation, while S1P and pERK became more influential later.

Figure 7B shows the total-order response, which indicates each species’ direct and indirect effects on regulating the PbI. Comparing the total-order to the first-order responses showed consistency in species influence in most cases. However, the overall effects of S1P and pERK given stimulation through VEGF or VEGF combined with HGF led to a higher total-order response than the first-order response at later time points. This indicated that VEGF stimulation increased the indirect influence of S1P and pERK on the PbI. Observing the Sobol index of each species highlighted the more significant influence of NO under VEGF stimulation when compared to HGF stimulation. It allowed us to observe that PAK1_thr_ is the overall key regulator of PbI for both single and combined stimulations, given the definition used for the PbI.

### Complex interactions between VEGF and HGF: Implications for vascular permeability

**Figure 8:**
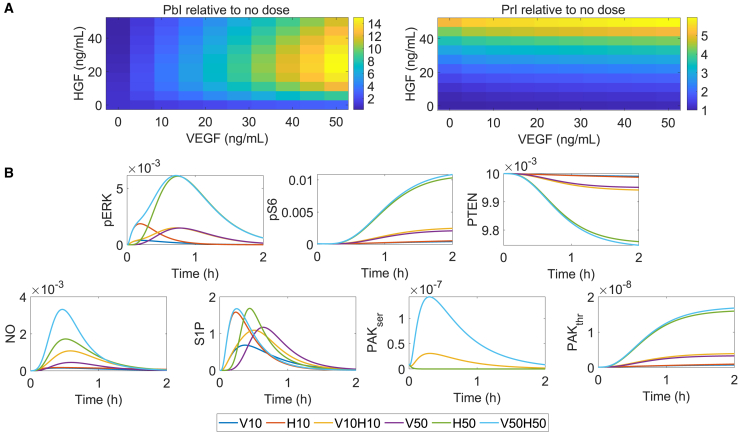
Dose response of PbI and PrI induced by VEGF and HGF showing a higher sensitivity of the PrI to HGF than to VEGF and a dose-dependent response of PbI to the growth factors under hypoxia (A) Heatmaps of PbI and PrI relative to a no-stimulation condition predicted for 1 h after stimulation. Intensity values are in arbitrary units. Dose combinations are shown on the *y* axis (HGF) and *x* axis (VEGF). (B) Time courses of species included in the equations for PbI and PrI given stimulation by VEGF, HGF, or both at 10 or 50 ng/mL, as indicated by colors in the label.

To further understand the dependency of permeability and proliferation on given combinations of VEGF and HGF, we simulated the dose response of the PbI and PrI, ranging from 0 to 50 ng/mL each. Figure 8 displays the results of our dose simulations.

Figure 8A shows the PrI change relative to a no-stimulation condition (VEGF = HGF = 0 ng/mL). As expected from our previous knowledge regarding the effects of VEGF and HGF, we saw a higher proliferation given the combination of higher doses of VEGF and HGF. Also, the results indicated that increases in HGF led to a more proliferative response than increases in VEGF. Figure 8B shows the temporal effect of stimulation by VEGF and/or HGF at 10 or 50 ng/mL on the species related to PrI. In agreement with what has been reported in the literature, HGF stimulation has a higher effect on increasing pERK and pS6 than VEGF,^34^ which is also apparent in the combined stimulation. Similarly, HGF led to a slight decrease in PTEN levels compared to the reduction seen under VEGF stimulation alone. Such responses explain the increased proliferation under HGF stimulation compared with VEGF stimulation.

Figure 8A shows our simulation of the response of PbI to single or combined doses of VEGF and HGF. The heatmap indicates that PbI increased with higher VEGF doses and decreased with higher HGF doses, as expected. We saw a maximum permeability at the combination of 50 ng/mL of VEGF and 25 ng/mL of HGF, indicating that their combination might lead to an increased PbI despite the reported anti-permeability effect of HGF. However, below and above this HGF dose, we saw a decrease in permeability for the same VEGF dose (50 ng/mL), indicating a complex interplay between the two factors. Interestingly, VEGF showed a potential to counteract the anti-permeability effect of VEGF at higher doses, and more HGF might be required to limit the PbI. The contributions of VEGF and HGF to defining the PbI and PrI were also time dependent, as shown in Figure 8B.

Our analysis showed an unexpected contrasting relationship between HGF stimulation and proliferation control, and to better understand it, we simulated the time response of the species included in the PrI equation (pS6, PTEN, and pERK) to a 20% increase in the value of kf Gab1SHP2, kf HGFMet, or kf Gab1PI3K. We compared it to the response simulated with the original value of the parameters, as presented in Figure S1.

We simulated the time response of the species included in the PrI equation (pS6, PTEN, and pERK) to a 20% increase in the value of kf Gab1SHP2 (gray dashed line with diamonds), kf HGFMet (gray dashed line with squares), or kf Gab1PI3K (gray dashed line with circles). We compared it to the response simulated with the original value of the parameters. The results indicated that increasing kf Gab1SHP2 increased the response of PTEN and decreased the response of pS6, which would lead to a decrease in PrI. Increasing kf HGFMet increased pS6 and pERK response and reduced PTEN response, compared to their baselines (solid black lines), indicating that this parameter would cause an increase in the PrI. Also, increasing kf Gab1PI3K by 20% of its original value would lead to a decrease in PTEN and an increase in pS6, which would also increase the PrI. Hence, while the PRCC evaluation indicated that kf Gab1SHP2 was positively related to PrI and kf HGFMet and kf Gab1PI3K were negatively associated with PrI, the time response simulated by increasing each of these parameters while maintaining the others at their original values indicated that all three parameters were positively associated with PrI. As PRCC measures linear or monotonic relationships while controlling for other variables, our results indicated a potentially non-linear relationship between kf HGFMet, kf Gab1PI3K, and kf Gab1SHP2 and the PrI. Also, it is possible that when considering the combined effects of all parameters, these three parameters’ partial effects on PrI are affected, which was not apparent in the analysis with PRCC, as PRCC controls for the effects of other parameters. This model result highlights the complexity of the network of reactions evaluated.

In short, consistent with our sensitivity analyses, determining an optimal combination of VEGF and HGF that increased proliferation while limiting permeability required careful consideration of these factors’ time and dose effects, given their complex interactions. Additionally, our dose-dependent simulation indicated that HGF led to a permeability and proliferative response independent of VEGF, as seen in the scenario of minimum VEGF with increasing HGF, in which we saw changes in the PbI and PrI.

## Discussion

In the past decade, HGF has been studied and proposed as a powerful pro-angiogenesis factor to treat conditions characterized by compromised blood flow. Dual therapy based on the combination of VEGF and HGF as a pro-angiogenesis therapy has been investigated as a treatment for different conditions characterized by ischemia.^21^^,^^22^^,^^65^^,^^66^^,^^67^ While both factors promote angiogenesis, HGF stimulates vascular stability and has anti-inflammatory and anti-necrosis properties beneficial for treating ischemic diseases. In the present study, we aimed to deepen our mechanistic understanding of the complex relationship between VEGF and HGF, their influence on their shared downstream signals, and their independence from each other and to investigate how these factors can be combined to achieve improved angiogenesis (i.e., increasing proliferation while maintaining vascular stability).

For that, we designed a computational model that included different signaling pathways involved in the HGF/VEGF signaling to regulate proliferation and vascular permeability, including a potential mechanism via differential regulation of PAK1 residues by VEGF or HGF, based on findings reported for HUVECs and pulmonary ECs.^53^^,^^55^ We carefully screened the literature for experimental data to calibrate our model and performed a technical evaluation of the model’s structural and practical identifiability analyses (based on the model structure and the data found in the literature for calibration). We optimized our model calibration using particle swarm optimization, a well-established global optimization technique, to define parameter values that led to good reproduction of experimental results both under normoxia (through HGF and/or VEGF stimulation at different doses) and under hypoxia. Next, we applied the Runs test in our fitting results, which indicated goodness of fit for our optimization. Additionally, we validated our model by testing its predictions against a separate dataset, which confirmed our initial calibration results, showing good agreement between model simulations and experimental data from the literature. Due to different reported results regarding HGF expression under hypoxia conditions (discussed later), we also performed an experimental analysis through ELISA of its expression in HUVECs subjected to low-oxygen conditions, which showed us a fast decay in HGF expression, as reported in Tables S8 and S9. We calibrated our model accordingly to reproduce our results. To support our findings, we quantified uncertainty by including confidence intervals based on our fitting results, which indicated good model calibration. Our methodological strategy agreed with good modeling practices, following the steps we presented in a previous work.^42^

Interestingly, while VEGFR2-driven activation of ERK/mitogen-activated protein kinase is well established as a key driver of endothelial proliferation and is typically the focus of computational models of angiogenic signaling, our sensitivity analyses suggest that VEGFR2-mediated mTORC1 activation via the PI3K-Akt-mTORC1-pS6 axis may also play a substantial role in promoting proliferation under hypoxic and normoxic conditions. Early work by Viñals et al. demonstrated that the PI3K-Akt-mTOR-S6K1 pathway was functionally essential for EC proliferation, showing that inhibition of p70 S6K effectively blocked EC proliferation. This reinforces the biological relevance of including pS6 in our model’s proliferation index.^69^ In addition, Mori et al. described an alternative mechanism in which mTORC1 inactivation led to FoxO3a nuclear accumulation, upregulating cell cycle inhibitors (p27 and p21) and suppressing proliferation.^70^ This indicates a potential regulatory impact of mTORC1 beyond pS6, involving transcriptional control via FoxO3a. Together, these studies support our modeling approach and highlight the importance of VEGFR2-mediated mTORC1 signaling in driving EC proliferation. Further experimental studies would help clarify the contribution of mTORC1 signaling to EC proliferation, particularly in the context of VEGF stimulation. Additionally, we have indications of which species could be targeted in pro- or anti-angiogenic therapies by determining which parameters are the major influencers of the PbI and PrI at different initial stimulations or oxygen conditions. For instance, our simulations indicated that Ca- and NO-related parameters were major influencers of PbI under normoxia given HGF stimulation. In contrast, Akt-related parameters were the major influencers of PbI given VEGF stimulation. Under hypoxia, we found different influential factors, such as parameters in the HGF pathway. Therefore, based on the condition and therapeutic strategy being studied, our findings could help guide further studies on HGF and VEGF and their interactions.

Pro-angiogenesis therapies involving VEGF are often affected by increased permeability and proliferation, both downstream effects of VEGF signaling. Studies on HGF have shown its potential to promote proliferation while constraining permeability (increasing vascular stability). To understand the individual and combined effects of VEGF and HGF on permeability and proliferation, we first identified the main parameters involved in regulating the PbI and PrI given single or combined stimulation by these growth factors. For that, we used PRCC to perform global sensitivity analysis and set the dose of HGF and VEGF accordingly (either at 0 ng/mL, indicating a minimal stimulus, or 50 ng/mL, indicating a higher stimulus). Our analyses evaluated the effect of differential stimulation of the system and the impact of oxygen limitation on defining which parameters were most influential in regulating proliferation and permeability. Our evaluation brought to light that the parameters’ influence on permeability changes with time (increasing or decreasing, after the initial model stimulation through VEGF and/or HGF) and depends on which stimulation was applied (VEGF and/or HGF). We find that, compared to other definers of PbI regulation, NO was a major regulator given VEGF stimulation and could, therefore, be targeted for permeability control. However, given HGF stimulation, the influence of NO on the PbI was not as high as NO's influence given VEGF stimulation.

Interestingly, given the combined stimulation with VEGF and HGF, we also found NO to have a high influence on the PbI, as in the case of VEGF stimulation. This indicated a complex interaction given dual stimulation that can be considered when evaluating strategies to induce angiogenesis while limiting vascular leakiness. Our results also indicated that PAK1_thr_ was highly important in regulating the PbI, regardless of the stimulation, and its influence should be evaluated in future studies. Our findings through sensitivity analyses advocated for the temporal and stimuli-dependent permeability control in ECs while indicating the importance of the PAK1 pathway on the diverging effect of VEGF and HGF. Our results allowed us to highlight key reactions that regulate both permeability and proliferation, which could be targets of therapeutic interventions (Figure 6), and to compare the effects of different species on the dynamical regulation of permeability (Figure 7), bringing attention to the PAK1 pathway for future experimental studies on HGF and VEGF signaling and therapeutic applications. We also found indications of a contrasting relationship between parameters downstream of HGF stimulation and their effect on the PrI (Figures 6 and S1). This relationship may stem from a potential non-linear relationship between kf HGFMet, kf Gab1PI3K, and kf Gab1SHP2 and the PrI. Also, it is possible that when considering the combined effects of all parameters, these three parameters’ partial effects on the PrI were affected, which was not apparent in the analysis with PRCC, as PRCC is affected by other parameters. This model result highlights the complexity of the network of reactions evaluated.

Considering some preliminary reports of the beneficial use of VEGF-HGF combined therapy in ischemic diseases,^21^^,^^34^^,^^65^ our goal was to better understand how these factors might affect vascular permeability and cell proliferation when combined. Additionally, we sought to investigate whether HGF’s effect requires VEGF signaling through VEGFR2, which remains an open question in the field. Therefore, we tested changes in PbI and PrI given different combinations of these factors. Our simulations suggest that vascular permeability was indeed modulated by both VEGF and HGF signaling under hypoxia, and their interaction produced non-linear, dose-dependent effects. While VEGF induced a dose-dependent increase in permeability, consistent with its established pre-permeability role, our results suggested that HGF can partially counteract this effect at certain intermediate doses, demonstrating its potential regulatory role under hypoxic conditions. However, our dose-based analysis (Figure 7A) also suggested that at certain VEGF doses, the pro-permeability effect dominated. It is important to reiterate that this finding was based on the species chosen to define the PbI and PrI and did not consider other aspects, such as inflammatory agents or the presence of pericytes. Regardless, it presents an important aspect of the complex interaction of VEGF and HGF and their permeability regulation, which could be tested in future studies to improve the efficacy of pro-angiogenesis and anti-permeability therapies. Additionally, our simulations indicated that HGF could alter EC permeability and proliferation independent of the activation of the VEGF-VEGFR2 pathway. Our model provides a useful framework for evaluating the distinct and combined roles of VEGF and HGF under early hypoxic conditions, which can help guide the design of more refined pro-angiogenesis therapies, aligning with recent efforts to promote angiogenesis while minimizing vascular leakage. These studies would also be useful in guiding anti-angiogenic and anti-permeability therapies such as those used in ocular diseases.

Our work seeks to provide insights into the complex interaction between VEGF and HGF and the consequences of their combination on regulating permeability and proliferation. The high number of species, parameters, and equations included in the model increases its complexity but brings it closer to biological scenarios. Future integration with experimental *in vitro* and *in vivo* studies will help us advance the work with these important growth factors and will further contribute to a better design of therapeutic strategies for pathological ischemic conditions. Given the required environmental considerations, we anticipate that our model can be applied to test the effects of therapeutic targets within those involved in the VEGF-HGF signaling. It can be applied to modeling different angiogenesis-dependent diseases such as PAD, cancer, and ocular diseases such as neovascular age-related macular degeneration. Additionally, it can be extended to incorporate new pathways and conditions to comply with specific scenarios being studied.

### Limitations of the study

Previous works in HUVECs and pulmonary ECs indicated potential Rac1 interactors that can modulate its influence on PAK1 phosphorylation, specifically, Tiam1 and Asef (both stimulated by HGF and leading to protective effects on the endothelial barrier) and Vav2 (stimulated by VEGF, leading to an increase in permeability).^53^^,^^62^^,^^63^^,^^64^ However, model calibration based on data obtained from different cell types can be a model limitation, and more studies focusing on Tiam1 and Asef activation by VEGF in HUVECs or microvascular ECs would be highly relevant to advance our knowledge in the field.

Previous works have also proposed different mechanisms through which VEGF and HGF differ in their action regarding vascular permeability. Although both factors work through Rac1 to activate PAK1, it has been shown that modulation of Rac1 interactors (Tiam1 and P-Rex1) stimulates guanine nucleotide exchange factor-specific signaling cascades.^68^ In this work, we followed this idea by proposing PAK1 activation at different residues by VEGF and HGF as an influencer of vascular stability. In this work, we established semiquantitative indices to represent changes in vascular permeability and cell proliferation based on changes in species concentration included in our model. Although these metrics did not offer a direct, fully quantitative indication of what to expect *in vivo*, they allowed us to gain an initial perspective on the changes in permeability and proliferation in a controlled environment compared to a no-stimulation condition. Understanding which reactions and factors contributed to regulating HGF/VEGF-induced vascular permeability and cell proliferation was essential to finding targets that enhance the positive effects desired therapeutically. Based on that, we performed global sensitivity analysis through PRCC on all model parameters under various initial conditions (changing stimulation through HGF, VEGF, or both, under normoxia or hypoxia), and we observed which parameters were most influential for regulating the PbI and PrI. It is important to note that our results are based on our definition of each index, and such a definition should be amended if other agents influencing permeability or proliferation are being considered. Our simulations showed that hypoxia had a greater effect on defining the major contributors to the PbI than the PrI, given combined stimulation by VEGF and HGF. This aspect could be further tested experimentally, as it would help us understand the pathology of ischemic diseases and tailor therapies to achieve the desired angiogenic effects.

While our model is the first mechanistic model regarding the relationship of VEGF and HGF and their effects on permeability and proliferation at a high level of detail in ECs, it can be further improved by including other aspects. For instance, the effects of hypoxia on the concentration of VEGFR2 and cMet, the effect of isoforms of VEGF, HGF, and VEGFR, and that of soluble receptors on downstream signaling could be important additions. The work of Fearnley et al. provides compelling evidence that different VEGFA isoforms (such as VEGF-A165, VEGF-A121, and VEGF-A145) can lead to distinct quantitative patterns of VEGFR2 activation, endocytosis, and degradation.^71^^,^^72^ In turn, these isoform-specific regulations shape endothelial responses by differentially modulating phosphorylation dynamics of Akt and ERK.^72^ Incorporating these dynamics into future mechanistic models would provide more accurate predictions of endothelial phenotypes under healthy and disease contexts (in which isoform ratios can be altered). Furthermore, differential receptor trafficking and degradation may alter cellular response, regulating permeability and proliferation in unique manners. In fact, previous studies have shown that stimulation with different VEGF isoforms led to structurally different vasculature and regulated pro- and anti-angiogenic signals differently.^71^^,^^72^^,^^73^ These findings highlight the need for models with a higher level of complexity and detail, expanding to isoform-specific frameworks, as well as the need for more temporal experimental data for specific conditions to be used for model calibration and validation. These could aid in explaining conflicting observations in the literature and support isoform-specific therapeutic targeting in diseases such as cancer and cardiovascular ischemia. Additionally, to improve the PbI metric, future work could incorporate signaling through TSP1 and the effect of other cell types, such as macrophages (for the study of inflammatory conditions) and pericytes (to study extracellular events related to vascular stability). Additionally, it is important to note that our simulations of the dose combination of VEGF and HGF considered the changes in PbI and PrI relative to the condition in which both HGF and VEGF are at zero. *In vivo*, this scenario would not occur, with a basal concentration of the factors being present. Our results represent changes seen given a very small amount of VEGF and/or HGF present compared to higher concentrations of either factor. Also, parameter tuning for unknown parameters was performed manually due to the limited quantitative experimental data available. Future studies with richer datasets could employ methods such as Bayesian optimization to refine parameter estimation and uncertainty quantification.

We also highlighted that during our search for data to define our model structure, we found that different results regarding the effect of hypoxia on HGF expression have been reported. Fan et al. reported an increase in HGF in HUVECs under hypoxia conditions (1% O_2_) using a hypoxic chamber.^74^ Hayashi et al. reported a decrease in HGF in human aortic ECs under hypoxia using an anaerobic device.^16^ Our experiments with HUVECs placed in a hypoxic chamber at 1% O_2_ showed a fast decrease in HGF levels, with a decrease of ∼86% of its initial concentration in the initial hour (measured through ELISA). It is worth noting that ELISA detects splice variants and intact versions of the targeted protein; therefore, our evaluation of HGF expression is for both. Potential explanations for the distinct results reported could be the cell type evaluated, the mechanism of hypoxia induction, and the different effects of hypoxia on HGF isoforms, which should be assessed in future studies. The results reported in this work for hypoxic conditions considered the calibration based on the data we collected (Tables S8 and S9; Figures S2 and S3). Additionally, we found different reports of a quantitative change in pAkt and pERK concentration in HUVECs under normoxia given single or combined stimulation with VEGF and HGF,^22^^,^^34^ which can be due to the initial amount quantified using different methods. Regardless, we could still observe the relative effect of combined or single doses of VEGF and HGF on downstream responses, such as proliferation and permeability, which qualitatively corresponded to what is reported in the literature. It is also worth noting that in the model, we assumed that the effects of hypoxia on HGF occurred through HIF signaling and regulation of cAMP (not included explicitly in the model), based on an early work.^16^ Additionally, while our work focuses on early endothelial responses (<2 h) under normoxia or hypoxia (1% O_2_), Ulyatt et al. reported a metabolic shift during sustained hypoxia (48 h), including VEGFR1 upregulation, downregulation of VEGFR2, and increased GLUT1 expression.^11^ These findings suggest a shift in VEGF receptor-mediated signaling and cellular metabolism over time. Our current model was developed to capture the acute phase of hypoxic signaling and did not include longer term transcriptional and metabolic adaptations. Incorporating metabolic feedback (such as AMPK-mTOR pathway crosstalk) in future modeling frameworks would be an important addition to explore how energy stress influences angiogenic signaling, proliferation, and permeability responses over extended periods of hypoxia.

## Resource availability

The authors confirm that the data supporting the findings of this study are available within the article and the supplemental information (Tables S1–S9 and S10; Figures S1–S3). The.sbproj main file is publicly available at https://doi.org/10.5281/zenodo.15428342.

### Lead contact

Requests for further information and resources should be directed to and will be fulfilled by the lead contact, Rebeca Hannah de Melo Oliveira (rdemelo1@jh.edu).

### Materials availability

This study did not generate new unique reagents.

### Data and code availability

The authors confirm that the data supporting the findings of this study are available within the article and the supplemental information.

• The computational model developed in this study is publicly available as a Zenodo file (the accession link is listed in the key resources table) in SBML and a SimBiology project format [.sbproj]).
• The datasets employed in this work for model calibration are available in each referred publication and can be found in the original papers. The data we collected for HGF expression under hypoxia are available as a supplemental information pdf file (Tables S8 and S9; Figures S2 and S3).
• All other related items related to the model (i.e., Observables, SIA, PIA, Reactions, Doses, Rules, ODEs, Species, Parameters, and Variants) are available in the supplemental information (Tables S1–S7) and excel files (Table S10). Any further data requests should be directed to the corresponding author.

## Acknowledgments

The authors thank members of the Popel laboratory, including Dr. Yu Zhang, for helpful comments and discussions, especially on the model methodology. We would like to dedicate this work to A.P., who sadly passed away during this research. His contributions and spirit will always be remembered. The work was supported by 10.13039/100000002NIH grants 5R01CA196701-08, 5R01CA237597-05, 1R21NS138938-01, 5R01CA138264-15, and 2R01EY028996-05A1 and a grant from the Lustgarten Foundation and a Graduate Fellowship from 10.13039/501100002322CAPES (Coordenação de Aperfeiçoamento de Pessoal de Nível Superior - Brasil)-Fulbright - Finance Code 001.

## Author contributions

R.H.d.M.O. and A.S.P. developed the concept of the study; R.H.d.M.O. constructed the model, performed all simulations, and wrote a draft of the manuscript; A.P. performed ELISA experiments on HGF and wrote the respective section in the manuscript; and A.P.P., B.H.A., and A.S.P. edited the manuscript.

## Declaration of interests

The authors declare no competing interests.

## STAR★Methods

### Key resources table

**Table:** 

REAGENT or RESOURCE	SOURCE	IDENTIFIER
**Biological samples**		

Human Umbilical Vein Endothelial Cells (HUVECs)	N/A	N/A

**Chemicals, peptides, and recombinant proteins**		

RIPA Buffer	Thermo Fisher Scientific	Cat# 89900
Hypoxia gas mixture (1% O_2_, 5% CO_2_, 94% N_2_)	Airgas	N/A
Phosphate Buffered Saline (PBS)	N/A	N/A

**Critical commercial assays**		

Quantikine Human HGF ELISA Kit	R&D Systems	Cat# DHG00B
DC Protein Assay	Bio-Rad	Cat# 5000112

**Experimental models: Cell lines**		

Human Umbilical Vein Endothelial Cells (HUVECs)	N/A	N/A

**Software and algorithms**		

Simbiology	MathWorks	MathWorks, R2024a
Grabit (MATLAB tool)	MathWorks File Exchange	N/A
ImageJ	NIH	RRID:SCR_003070

**Other**		

Simbiology project for model	Zenodo	https://doi.org/10.5281/zenodo.15428342
Hypoxia Incubator Chamber	STEMCELL Technologies	Cat# 27310

### Experimental model and study participant details

HUVEC were cultured until confluence in several T- 25 flasks, one for each time point: 1, 2, 4, 6, 8, 24 h, and a 0 h negative control. Hypoxia was simulated using a hypoxia incubator chamber (Stemcell Technologies, Cat# 27310), an airtight chamber that is flushed with a 1% O2, 5% CO2, 94% N2 gas mixture (Airgas) and placed in a standard 37°C cell culture incubator. Before the incubation, the culture media of each flask was exchanged with fresh media, and all flasks were transferred to the hypoxic chamber together, except for the negative control. At each time point, the corresponding flask was removed from the chamber and lysed using the following protocol. The cells were washed twice with fresh PBS, lysed with 500 μL RIPA buffer, incubated on ice for 10 min, scraped with a cell scraper, sonicated for 10 s at low power, centrifuged at 18,000 RCF for 10 min at 4°C, and the supernatant was collected and stored at −20°C. Once all the time points were collected, total protein quantification was performed on the lysates using the DC Protein Assay (Bio-Rad, Cat# 5000112) following the manufacturer’s protocol. Finally, the HGF concentration of each lysate was quantified using the Quantikine Human HGF ELISA Kit (R&D Systems, Cat# DHG00B) following the manufacturer’s protocol.

### Method details

#### Model structure: downstream pathways included

When building mechanistic models to investigate specific biological questions, a moderate level of complexity is needed to ensure accurate biological representation while avoiding issues with overparameterization and limiting computational costs during model optimization and simulation. With that goal in mind, we incorporated six pathways in this model, according to what is currently known regarding signaling in EC via VEGF and HGF. At several points, the pathways converge and interact with each other, as can be seen in Figure 1. Below, we describe in detail the pathways that we included.

##### The VEGF-VEGFR2 signaling pathway

VEGF (specifically, VEGFA) binds to its receptor (VEGFR2) on the surface of ECs. Being a dimer, VEGFA dimerizes VEGFR2 monomers, which results in receptor autophosphorylation at specific intracellular tyrosines (such as Y951 and Y1175).^75^ The phosphorylated tyrosines activate distinct downstream signals; for instance, pY951 induces Akt activation through PI3K (a mechanism of cell survival regulation), and pY1175 leads to the activation of ERK1/2 through PLC γ (which regulates cell proliferation) and of Akt.^76^ VEGF receptors are also internalized and recycled through ligand-induced VEGFR2 endocytosis or constitutive recycling of inactive receptors.^77^ Surface and internalized VEGFR2 can activate downstream signals. In the current model, we represented such interactions in a simplified form, including receptor interaction with VEGFA and its subsequent phosphorylation, internalization, recycling, and degradation (Figure 2A).

##### The HGF-cMet signaling pathway

ECs express cMet receptors on their surface, which are activated by binding to HGF. Upon HGF binding to the cMet receptor, the receptor dimerizes/oligomerizes, activates transphosphorylation of tyrosines (Tyr1234 and Tyr 1235) in the kinase domain, followed by additional phosphorylation of other tyrosines (Tyr 1349 and Tyr 1356) in the C-terminal regulatory tail.^78^ The second round of phosphorylation provides a docking site for multiple substrates of downstream signal transduction, such as Src, Gab1, and Gab2. Gab1 binds to the bipartite docking site (Y14, Y1349, Y15, and Y1356) of the activated c-Met receptor.^79^ Upon cMet activation, SHP2 is recruited to the phosphorylated receptor via SH2 domain and binds to Gab1 via SH3 domains, thereby stabilizing Gab1-c-Met associations in vivo.^8^^,^^79^ Gab1 acts as a docking protein, with tyrosine residues that can be phosphorylated to serve as docking sites for other proteins such as PI(3)K (through p85 subunit), PLC-γ, and Shp2.^51^ Gab1 binding the p85 subunit of PI3K removes its inhibitory action on the catalytic subunit p110 of PI3K.^80^ PI3K can then phosphorylate PIP2, forming PIP3, and proceed with its downstream signaling, leading to the phosphorylation of Akt. This process promotes EC migration and angiogenesis.^81^

Phosphorylated Gab1 also binds to PLCy, which hydrolyzes phosphatidylinositol-4,5-bisphosphate (PIP2) at the inner face of the plasma membrane and generates diacylglycerol (DAG) and inositol-1,4,5-trisphosphate (IP3).^82^ Phosphorylated Gab1 binding to SHP2 forms a complex that activates ERK1/2, which regulates cell migration.^51^ HGF-cMet interaction also recruits and phosphorylates Gab2, which competes with Gab1 for cMet interaction phosphorylation. Additionally, Gab2 endogenously inhibits the activation of ERK1/2 and AKT downstream of HGF/c-Met in the ECs.^51^ Figure 2B shows the HGF-cMet pathway we modeled.

##### The Ca^2+^-NO signaling pathway

Phosphorylated VEGFR2 and cMet stimulate PLC γ, which then catalyzes the hydrolysis of PIP2 into IP3, a regulator of calcium cycling dynamics. Figure 2C represents this process, which relates to the phosphorylation of eNOS. IP3 diffuses into the cell and binds to IP3-sensitive calcium release channels on the endoplasmic reticulum (ER).^83^ The binding leads to calcium release from the ER with a consequent decrease in calcium concentration in this compartment. This decrease causes calcium release-activated calcium channels (CRAC) to open, allowing extracellular calcium influx. The action of the surface membrane PM pump and ER SERCA channels balances calcium concentration in the cytosol.^84^

Under hypoxia, we included the activity of the sodium-calcium exchanger (Na+ − Ca2+) proposed by Berna et al.^13^ in our model. Hypoxia inhibits mitochondrial oxidative phosphorylation, which increases the cellular requirement of glucose for anaerobic glycolysis to replenish energy. The required glucose can enter the cell via a Na+-glucose-like co-transporter. The co-transporter increases the amount of Na+ in the cytosol, which the Na+ then balances − Ca2+ exchanger, taking Ca2+ in and Na+ out. Through this mechanism, hypoxia increases intracellular calcium concentration. In our model, we represented these mechanisms by a single reaction that depended on the concentration of HIFs and external calcium.

Calcium binds to calmodulin and then to eNOS, leading to its phosphorylation (a process that also depends on Akt phosphorylation, activated by Src).^10^ Additionally, Src activates the chaperone protein HSP90, which facilitates eNOS phosphorylation. Phosphorylated eNOS reacts with Arginine, converting it to Citrulline and producing nitric oxide (NO).^85^

##### The Akt signaling pathway

Figure 2D shows the Akt-eNOS signaling pathway that we modeled. The pathway is initiated by VEGFR2 phosphorylation (surface and internalized). Phosphorylated VEGFR2 leads to the activation of Src in a complex with TSAd, as presented in previous works.^10^^,^^47^ Subsequently, Axl-1 is phosphorylated by phosphorylated TSAD-Src and then undergoes a second auto-phosphorylation. The double-phosphorylated Axl-1 is recruited to the membrane, and it activates PI3K.^52^ Following this, PI3K leads to the phosphorylation of PIP2 to form PIP3 (a process that PTEN can reverse). PIP3 then recruits Akt and PDK1 to the cell membrane. Following, mTORC2 kinase acts on recruited Akt, leading to its phosphorylation on serine 473 residue.^86^ Then, the recruited PDK1 phosphorylates Akt on its threonine 308 residue. Akt activity is also upregulated under hypoxia, which we assume is mediated by the HIF1/2-VEGFA signaling.^87^

Akt activation stimulates mTORC1 by phosphorylating and inhibiting the activity of tuberous sclerosis complex 2 (TSC2) and PRAS40, which are negative regulators of mTORC1 activity.^88^ mTORC1 activation leads to the phosphorylation of various substrates, such as S6 kinase 1 (S6K1). S6K1 phosphorylates ribosomal protein S6 (RPS6) and other targets related to cell cycle regulation. RPS6 stimulates the protein synthesis required for cell growth and proliferation (such as components in the G1 phase like cyclin D1 and CDK2).^69^

##### The ERK signaling pathway

The phosphorylation of VEGFR2 also activates the Raf-MEK-ERK pathway, as shown in Figure 2E. In this pathway, PLC-γ is activated by pVEGFR2 and leads to the generation of DAG and IP3.^75^ DAG binds to PKC and calcium, activating and phosphorylating Raf, MEK, ERK, and sphingosine kinase 1 (SphK). Activated IP3 stimulates calcium release from the endoplasmic reticulum, and the released calcium can bind to calcium and integrin binding protein 1 (CIB), forming a complex. Active SphK (pSphK) is translocated to the plasma membrane by CIB, and a complex is formed between Ca-CIB-pSphK. SphK1 will then phosphorylate its ligand (Sph), forming diffusible sphingosine 1 phosphate (S1P).^9^ Finally, S1P activates Ras, forming RasGTP, which stimulates Raf phosphorylation. As previously mentioned, we modeled the increase in ERK1/2 under hypoxia as mediated by the increase in VEGFA transcription given HIF stabilization under hypoxia.^89^

##### The HIF signaling pathway

Under normoxia, oxygen molecules form complexes with Fe-DG-PHD2/PHD3, leading to HIF1/2-α hydroxylation and subsequent polyubiquitination by VHL (von Hippel-Lindau ubiquitin E3 ligase), which marks them for degradation. Finally, HIF undergoes proteasomal degradation.^36^^,^^90^ However, under hypoxia, both PHD activities are reduced, and the hydroxylation of HIF1/2-α does not occur. Under hypoxia, HIF1/2-α are stabilized and move to the nucleus, where they dimerize with HIF1β. The newly-formed complex then binds to the hypoxia element site on DNA, leading to the expression of different genes as a response to hypoxia, along with VEGF, erythropoietin (EPO), and glycolytic enzymes.^12^^,^^91^ HIF isoforms are also regulated by FIH, but we do not focus on this reaction in our model, as was done in previous work.^36^ Additionally, hypoxia downregulates HGF in vascular ECs, a mechanism mediated by adenosine 3′,5′-cyclic monophosphate (cAMP) regulation.^16^ In our model, we consider this mechanism to be modulated by HIF signaling, as HIF has also been reported as a down regulator of cAMP.^92^ For simplicity, we did not include cAMP in the model, but the effects of hypoxia on HGF were modeled through a Hill equation with a Hill coefficient of 4 to account for the fast decrease in HGF observed experimentally.

We built a simplified model of the HIF pathway (Figure 2F) based on several other models from the literature.^37^^,^^93^^,^^94^ Our model considered oxygen (O2) levels to be constant (i.e., not consumed by the reactions). We included the two primary forms of HIF described in hypoxia-induced angiogenesis, HIF1 α and HIF2 α (HIFs, in general) which undergo similar processes. We considered the effect of PHD2 and PHD3 as regulators of HIF signaling. PHD2 inhibits both HIFs and is upregulated by HIF1 α. The two HIFs upregulate the activity of PHD3. HIF2 is inhibited by both PHDs. PHDs inhibition of HIFs depends on O_2_ concentration (more inhibition for normoxia, less for hypoxia).^94^ Additionally, basal rates of production and degradation of HIFs mRNA and protein are included in our model.^93^ Finally, we modeled the binding to HIF1 β (under hypoxia) of both HIFs and the downstream upregulation of VEGFA mRNA and protein due to hypoxia.^37^ We considered the amount of HIF1 β to be constant in the model. Our modeling strategy allowed us to reproduce the HIF response to different O_2_ levels as observed in the time-courses from experimental data.

To calculate the molar concentration of O_2_ to include in the model, we employed the oxygen solubility in water at 37C of ∼1.3 μM/mmHg.^36^^,^^95^ The oxygen partial pressure under normoxia for HUVECs was considered to be similar to the environment used in *in vitro* experiments (i.e., 21% O_2_ ≈ 160 mmHg ≈ 209 μM) and under hypoxia (i.e., 1% O_2_ ≈ 8 mmHg ≈ 10 μM).

##### The HGF/VEGF-PAK1 signaling pathway

In HUVECs stimulated with VEGF, PAK1_ser141_ is phosphorylated during the activation of Rac1.^96^ Meanwhile, in pulmonary ECs, HGF treatment has been shown to cause PAK1 autophosphorylation at threonine 423, leading to endothelial barrier recovery and protective effect through activation of cytoskeletal and cell adhesion-associated effectors of Rac that enhance adherens junctions and peripheral actin cytoskeleton.^61^ Based on these findings, we included PAK1 regulation in the model, hypothesizing that the opposite effect on permeability would be observed given stimulation by VEGF and HGF. To account for this effect and based on the availability of data to calibrate this section of the model, we incorporated PAK1 signaling induced by these factors in the model structure. PAK1_ser141_ phosphorylation by VEGF is mediated by activation of the TSAD-Src complex, which is not influenced by HGF. Meanwhile, PAK1_thr423_ phosphorylation is mediated by PI3K activation, which is downstream of both HGF and VEGF and therefore influenced by both factors. Previous work has indicated that upon activation of Src by VEGF, Rac1 undergoes a biphasic activation through Vav2, followed by PAK1_ser141_ activation.^62^^,^^64^ This leads to the internalization of VE-cadherins through β arrestin2 and a sequential increase in permeability. Also, previous work reports that PI3K activation by HGF leads to activation of Tiam1 and Asef, which induce Rac1 activation followed by PAK1_thr423_ phosphorylation.^53^^,^^61^ This event enhances adherens junctions and peripheral actin cytoskeleton, limiting permeability. For simplicity, we did not explicitly include all mediators of activation of PAK1 in the model and focused on the activation of TSAD-Src and PI3K mediating PAK1 activation (Figure 2G).

#### Model structure: Parameterization

The model included a simplified version of VEGF-VEGFR2 signaling, with receptor internalization and phosphorylation, and a state variable representing the mRNA VEGFR2 (regulated by VEGF and HGF stimulation). We included HGF signaling to its receptor, cMet, leading to the downstream activation and phosphorylation of Gab1. The pathways were connected by their downstream effects on ERK and Akt activation. Additionally, we included PAK1 activation mediated by the phosphorylation of Src and PI3K as influencers of vascular permeability. Of the initial 186 parameters, after searching the literature, 45 values were unknown, and 141 were obtained from previous data in the literature. Parameters obtained from the literature were selected from previous computational models and/or reports focused on ECs, for consistency. We initially focused on data available for HUVECs, and then expanded the search for ECs in general, due to data limitations. When association (kon) and dissociation (koff) rates were not directly available from the literature, but the dissociation constant (Kd) was, we estimated the dissociation rate and calculated the association constant rate based on Kd = koff/kon. We prioritized the use of experimental data wherever possible or used values from peer-reviewed computational models with similar pathway architecture. For the unknown parameters, we set the initial values before the model optimization through manual-tuning, and their values are considered model assumptions. The final species' initial concentration and parameter values are included in supplemental information (Tables S6 and S12).

### Quantification and statistical analysis

#### Structural and practical identifiability analyses

Before fitting our model to obtain the values of unknown parameters, we performed identifiability analyses to select the subset of parameters that can be uniquely identified based on the model structure and the available experimental data that will be used for fitting. For Structural Identifiability Analysis (SIA), we employed the MATLAB®-compatible toolboxes GenSSI 2.0^97^ and STRIKE-GOLDD 4.0 (ProbObsTest).^98^ GenSSI employs symbolic power series expansion methods, being efficient for models of moderate size. Meanwhile, the ProbObsTest algorithm from STRIKE-GOLDD provides a more scalable and computationally efficient SIA investigation for larger rational models, by avoiding full symbolic computation of Lie derivatives. By using both, we ensured robustness of our SIA conclusions without being limited to the specifics of a single method. We determined the unknown parameter to be structurally identifiable (SI) if it was marked as identifiable by at least one of the toolboxes. We then selected the final list of unknown structurally identifiable parameters by including parameters that passed the SIA test on at least one of the tools. The SIA results obtained with GenSSI and STRIKE-GOLDD are presented in Figure 3A (identifiability tableau generated by GenSSI and the asterisk marks the STRIKE-GOLDD result).

Next, we performed a practical identifiability analysis (PIA). PIA is often used as a post-fitting analysis, indicating the trustworthiness of predicted parameters, but can also be pre-evaluated based on two principles: the parameters must be influential to the observables, and the parameters should not be collinear (i.e., with effects that changes in other parameters may compensate).^99^

Based on these evaluations, we first performed a visual collinearity evaluation of the selected subgroup of structurally unknown parameters. Our evaluation consisted of removing potential collinearities, for example, the rate of phosphorylation and the rate of dephosphorylation in the same reaction, or the Michaelis constant and the maximum rate achieved by the system (in a reaction modeled following Michaelis-Menten kinetics). This contrasting action indicated that parameters might compensate for each other’s effects, therefore being classified as collinear. Following this strategy, if we identified collinearity, we removed one or more (if three or more are collinear) of the collinear parameters to avoid the issue of effect compensation before fitting.

Following collinear removal, we evaluated the influence of our SI unknown parameters on the set of observables collected from the literature. We employed global sensitivity analysis through Partial Rank Correlation Coefficient (PRCC) on all unknown SI parameters and evaluated their effects on each observable.^100^ We varied the parameter values one order of magnitude above and below the estimated initial values. If the parameter presented a PRCC ≥0.1 for at least one of the observables, we considered it influential and included it in the subgroup to fit. The minimum value of 0.1 was chosen to allow for more parameters to be included in the fitting process and is an assumption of the PIA sensitivity analysis we conducted. We set the number of samples as the number of parameters ∗100. Therefore, here, practical identifiability was preliminarily assessed prior to fitting, following the approach of Gábor et al. (2017), by combining global sensitivity analysis and collinearity evaluation. Parameters showing sufficient influence and low collinearity were selected for fitting.

The supplemental information (Tables S2 and S3) show the parameters evaluated in each step. We then proceeded with model fitting.

#### Global optimization with particle swarm optimization

We collected data from literature focusing on western blots or ELISA samples for global optimization. These *in vitro* data were collected for human umbilical vein endothelial cells (HUVECs) and contained both time and dose responses to VEGF, HGF, and hypoxia. To reduce the computational cost, we separated the fitted parameters into two groups: parameters related to hypoxia and parameters related to normoxia. As the HIF signaling pathway module included in the model is upstream of the VEGF and HGF pathways, we first calibrated the parameters in the HIF pathway and then the parameters in the downstream pathways. After the first round of fitting (for parameters in the HIF pathway), we set the calibrated parameters to their new optimized values. Then, we perform the second round of fitting for the remaining parameters. The time course data were normalized to the maximum reported in the experiment before fitting, and the equivalent procedure was done for the observables. We normalized the data to the initial value reported in the experiment for the dose-response of pAkt and pERK to HGF, VEGF, and VEGF + HGF. We estimated the initial values of pAkt and pERK in the model to better reproduce the experimental results. Western blot data were evaluated using ImageJ to extract relative activation values, and densitometry data were evaluated using the tool Grabit available on MATLAB®.

We implemented pooled Particle Swarm Optimization in SimBiology for model fitting, grouping the experimental data by time course or dose-response. We set the range of variation for the parameter values to be one order of magnitude above or below the initial value established by hand-tuning, and we obtained the predicted values that better matched the time and dose responses reported in the literature. Our post-fitting results compared to experimental data points are presented in Figure 4. The values found for each parameter are listed in the supplemental information (Table S12).

Additionally, to evaluate the goodness-of-fit, we employed the Runs Test of randomness using the function *runstest()* on the residuals calculated by the fitting process.^101^ Residuals were calculated as the difference between the experimental data values and the corresponding model-predicted values at each measurement time point, to represent the fitting error at each point. The Runs Test tests the hypothesis that the residual values are in random order at a default 5% significance level (null hypothesis). This test returns two values: h (representing the hypothesis test result) and p (representing the probability of finding a test statistic as extreme as or more extreme than the observed values under the null hypothesis. Results that show h = 0 would not reject the null hypothesis of randomness, and small values of p indicate doubt on the validity of the null hypothesis. Therefore, we performed the Runs Test for each fit obtained (for each observable), with the goal of obtaining h = 0 and higher values of p (>0.05). A *p*-value greater than 0.05 indicates a 5% probability that the observed runs could occur if the data were random, which we use as the cut-off for our Runs test.

#### Uncertainty quantification: 95% bootstrap confidence intervals

To evaluate the quality of the predictions of our fitted model, we computed 95% Bootstrap confidence intervals (CIs) using the “sbiopredictionci” function in MATLAB®’s SimBiology toolbox, specifying 100 of the predictions with 100 bootstrap samples. In short, the procedure involves resampling the residuals from the model fit with replacement, followed by refitting the model to each resampled dataset and simulating the model outputs. The 95% CIs were then determined by taking the 2.5th and the 97.5th percentiles of the simulated predictions at each time point. We then evaluated whether the experimental data points fell within the CIs and followed the time and dose trends seen experimentally. The results are presented in Figure 5.

#### Proliferation and permeability indices

Cell proliferation and vascular permeability (also referred to as leakiness and stability) are highly regulated processes under HGF and VEGF stimulation. Several considerations are needed to design a mathematical model to represent these aspects. In a previous work on a PAD model,^43^ our group implemented leakiness as a score where 1 represents non-leakiness and lower values represent increased leakiness seen under pathological conditions such as acute/gradual hindlimb ischemia. Additionally, EC proliferation was modeled as a function of VEGF165a availability. Another study evaluated EC hyperpermeability as a function of VEGF, histamine, and thrombin.^102^ EC hyperpermeability is characterized by myosin light chain (MLC) activation downstream of calcium (Ca^2+^) upregulation, which induces dissociation of EC-EC junctions by promoting cytoskeleton contraction. Additionally, *in vivo* and *in silico* experiments have shown that S1P regulates microvascular permeability by protecting the endothelial surface glycocalyx in microvessels.^103^ Mechanochemical aspects have also been recently modeled as influencers of EC permeability but, for simplicity, are not considered in our work.^104^ In the present work, the model is based on ordinary differential equations (ODEs) and describes the intracellular signaling dynamics in endothelial cells. Spatial aspects of endothelial cell permeability, such as explicit modeling of local heterogeneity, were not represented.

As our goal was to use our model to observe the effects of VEGF and HGF on permeability and proliferation of ECs through modulation of distinct pathways, to quantify such effects, we propose and present our definition of relative permeability and proliferation indices based on downstream-regulated species cited in the literature as regulators of either permeability, proliferation, or both. We employed a semi-quantitative strategy to observe changes in permeability and proliferation, which is described in this section.

We first established a permeability index (PbI) to evaluate the changes in EC and vascular permeability, as shown in Equation 1.

(Equation 1) $$PbI=\frac{\left(NO*PAK_{ser}\right)+k_{2}}{\left(S1P*pERK*PAK_{thr}\right)+k_{2}}$$

The index considers species included in the model instead of an explicit inclusion of junctions between ECs and is described by the relative amount of nitric oxide (NO), $PA{K1}_{ser}$, S1P, pERK and $PAK1_{thr}$. There is also evidence that Src activation downregulates junctions (VE-cadherin), promoting permeability.^105^^,^^106^^,^^107^ Based on our model structure, we considered the effects of Src to occur through the values of NO. The activation of PAK1 at different residues was also included, as it has been investigated as a potential way via which VEGF and HGF regulate permeability.^61^^,^^63^^,^^64^^,^^108^ ERK phosphorylation in HUVECs has been shown to regulate permeability by activating TGF β and suppressing eNOS.^109^ We included a scaling factor $k_{2}$ which is set to the smallest product of the species included in the calculation of PbI. Setting k to the smallest product ensures that it is relevant to the magnitude of the species involved, avoids the issue of division by zero, and maintains the proportional differences between numerator and denominator, allowing us to achieve a better semi-quantitative representation of changes in PbI. By including $k_{2}$, we set the baseline of PbI at 1 (when at least one of the species included in the nominator and denominator assume value 0).

Similarly, we established an index to quantify cell proliferation stimulus (PrI), considering regulators used as species in our model. The activation of ERK and Akt has a synergistic effect on promoting cell proliferation. Downstream of Akt activation, mTORC1 activation of S6K1 has also been related to increased cell proliferation.^69^^,^^70^^,^^110^ S6K1 activation leads to the phosphorylation of S6, thus, we include pS6 in our calculation of proliferation. Additionally, Src is a key inducer of cell proliferation downstream of VEGF and HGF stimulation.^111^ To limit the accumulation of effects, we define the index based on ERK phosphorylation^112^^,^^113^ and on S6 phosphorylation^69^ (which is downstream of Src, Akt, and mTORC1 activation). PTEN is a regulator of the Akt activation downstream of PI3K and, therefore, a negative regulator of the Akt-induced cell proliferation stimulus.^114^ Once again, we included a scaling factor, $k_{4}$, of the same magnitude as the smallest product, which better reflects changes in involved variables, providing more meaningful results. Including $k_{2}$ and $k_{4}$ is a deliberate methodological choice to improve our interpretability and sensitivity of the indexes. Equation 2 shows the relative index to estimate the proliferation stimulus.

(Equation 2) $$PrI=\frac{\left(pERK*pS6\right)+k_{4}}{\;PTEN+k_{4}}$$

Given the semi-quantitative approach we took with the definition of PrI and PbI, we used our simulations to evaluate the individual and combined effects of HGF and VEGF on permeability and proliferation. We assessed the various stimulation effects by comparing the values of PrI and PbI under stimulation by HGF and VEGF to a no-stimulation condition. Therefore, our baseline for comparison is the time simulation of PrI and PbI at a zero input of VEGF and HGF. The PbI and PrI indices were evaluated at specific time points depending on the analysis. For PRCC sensitivity analysis and hypoxia/normoxia comparisons, indices were calculated at one hour after stimulation. For Sobol sensitivity analysis and the dose-response heatmaps, indices were calculated at two hours after stimulation.

Our analysis showed an unexpected contrasting relationship between HGF stimulation and proliferation control, and to better understand it we simulated the time-response of the species included in the PrI equation (pS6, PTEN, and pERK) to a 20% increase in the value of kf Gab1SHP2, kf HGFMet, or kf Gab1PI3K. We compared it to the response simulated with the original value of the parameters. A detailed explanation is provided in the Figure S1.

#### Global sensitivity analyses

After model validation, we employed PRCC and Sobol sensitivity analyses to evaluate the influence of parameters and species included in the model on the PbI and the PrI. PRCC is a computationally-efficient rank-based method that quantifies monotonic influences of parameters on model outputs, enabling us to screen for parameter importance. In contrast, Sobol sensitivity analysis, is a variance-based method that decomposes output variance into first-order effects (contributions from individual parameters) or total-order effects (interactions between parameters). Combining PRCC and Sobol enabled us to balance computational efficiency with a more detailed characterization of species influence and interaction effects. We used the algorithm by Marino et al.^115^ to perform PRCC, varying the parameter values one order of magnitude above and below the post-calibration estimated values. During model development, PRCC was first used as part of the practical identifiability analysis (as previously mentioned) to select parameters for fitting. After the model calibration and validation, we performed additional PRCC analyses to explore how the influence of each parameter in the model varied under different conditions. This second PRCC analysis included varying all parameters in the model to quantify their influence on the PbI and PrI.

To evaluate the effects of the species included in our equation for the PbI, we used Sobol Sensitivity analysis^116^ (sbiosobol tool on Simbiology) and compared the sensitivity of the PbI to each species, stimulating the system with VEGF 10 ng/mL, HGF 10 ng/mL, or their combination for two hours of continued stimulation. Sobol Sensitivity analysis allows us to evaluate the direct (first-order) and indirect (total-order) responses of systems’ outputs to changes in inputs. The first-order response measures the direct effect of a single input on the output, ignoring interactions with other parameters. The total-order response evaluates the overall effect of a parameter, including its interaction with other parameters.

## Supplemental information

Document S1. Figures S1–S3 and Tables S1–S9

Table S10. List of all model reactions, ODEs and parameters, related to Figures 2, 3, 4, and 5 (A) List of model reactions. (B) List of model ODEs. (C) List of model parameters.
