# Supplementary material for: A mechanistic computational model of HGF-VEGF-mediated endothelial cell proliferation and vascular permeability
Source: iScience. 2025 Jul 24;28(8):113199. doi: 10.1016/j.isci.2025.113199 (PMC12355113; doi:10.1016/j.isci.2025.113199)
Supplement: Document S1. Figures S1–S3 and Tables S1–S9 [file mmc1.pdf]

iScience, Volume *28*

Supplemental information

A mechanistic computational model of HGF-VEGF-mediated endothelial cell proliferation and vascular permeability

Rebeca Hannah de Melo Oliveira, Akash Patil, Brian H. Annex, Arvind P. Pathak, and Aleksander S. Popel

Supplementary Material Figures

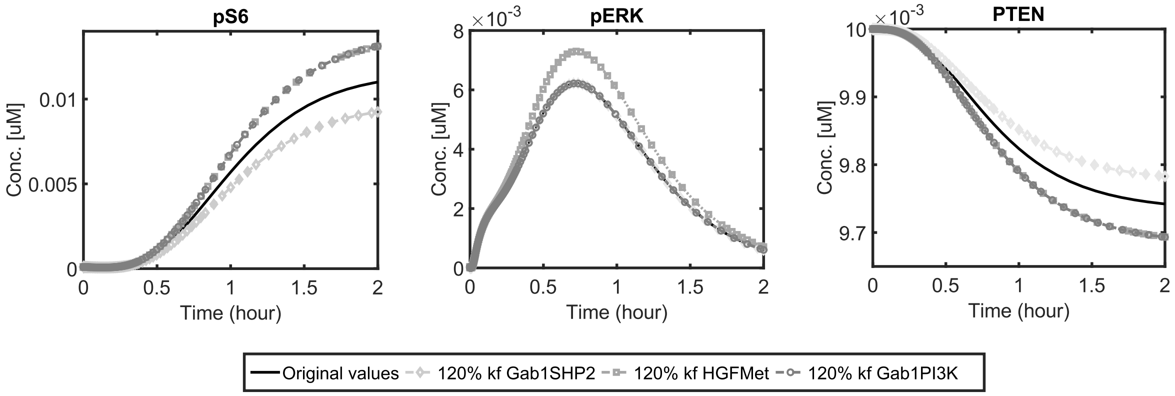

Figure S1: Time-response of pS6, pERK, and PTEN to increased kf Gab1SHP2, kf HGFMet, or kf Gab1PI3K. Solid lines represent the original value of the parameters. Related to Figure 8.

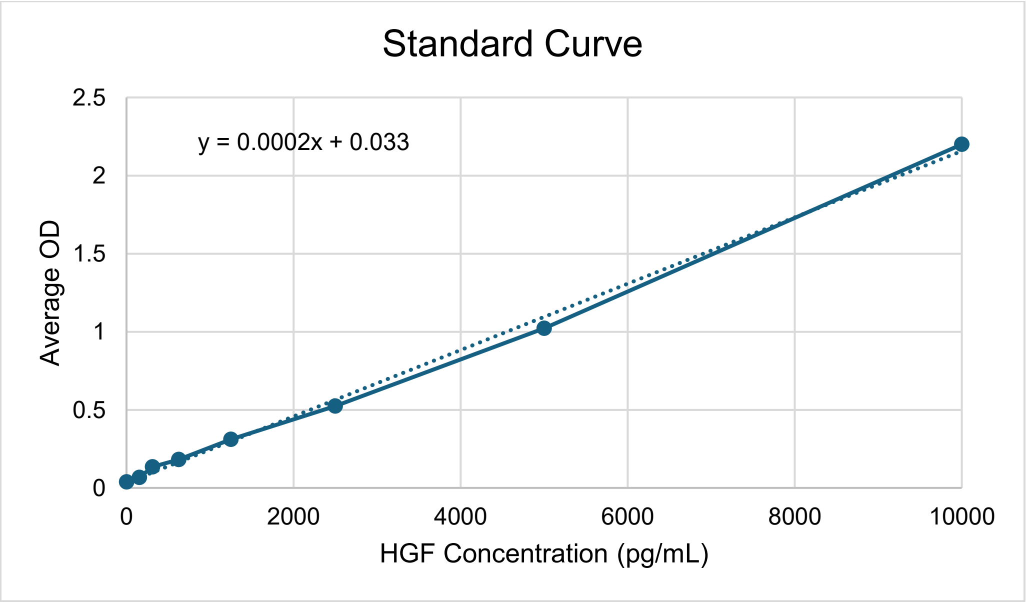

Figure S2: Standard curve for HGF quantification by ELISA. *The graph shows the relationship between known HGF concentrations (pg/mL) and the corresponding optical density (OD) readings. Each point represents the average OD for a given concentration. The linear regression line is shown as a dotted line, with the equation y=0.0002x+0.033y = 0.0002x + 0.033y=0.0002x+0.033, where* y *is the OD and* x *is the HGF concentration. This standard curve was used to interpolate HGF concentrations from experimental samples. Related to Figure 4.*

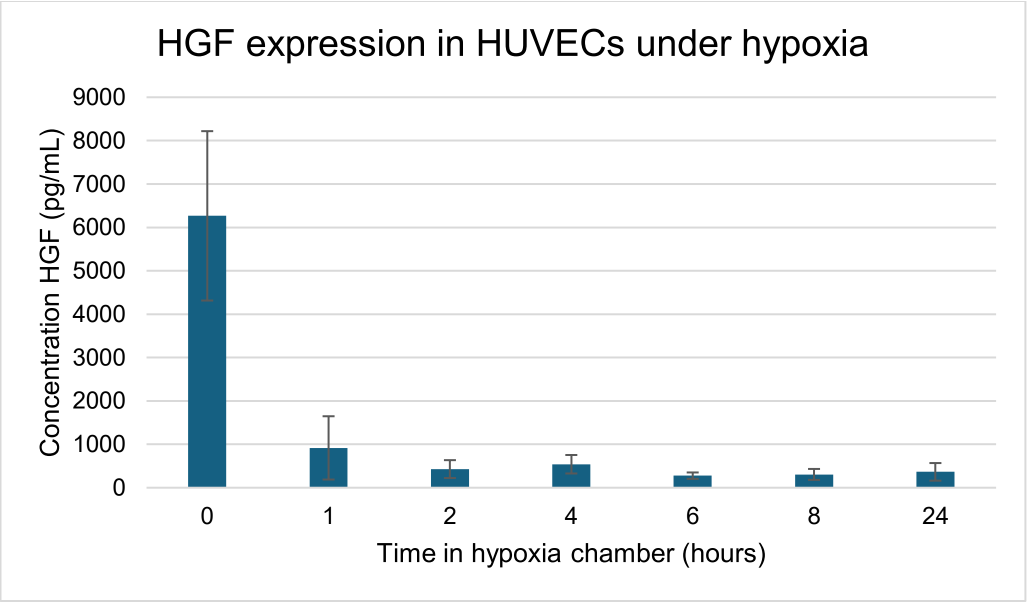

Figure S3: Time-course of HGF expression in HUVECs under hypoxia. *Human umbilical vein endothelial cells (HUVECs) were cultured to confluence and exposed to hypoxic conditions (1% O_2, 5% CO_2, 94% N_2) in a hypoxia chamber (Stemcell Technologies, Cat# 27310) for 1, 2, 4, 6, 8, and 24 hours. A 0-hour sample (normoxia) was used as a negative control. At each time point, cells were lysed in RIPA buffer, sonicated, centrifuged, and the supernatant was collected for analysis. Total protein content was quantified using the DC Protein Assay (Bio-Rad, Cat# 5000112), and HGF levels were measured using the Quantikine Human HGF ELISA Kit (R&D Systems, Cat# DHG00B). Results are presented as mean ± standard deviation (n = 3). A significant decrease in HGF concentration is observed after 1 hour of hypoxia, followed by sustained low levels through 24 hours. Related to Figure 4.*

Supplementary Material Tables

Supplementary table S1. Table of observables

Name	Expression
HIF2a_norm	c1.HIF2A_protein/max(c1.HIF2A_protein)
pR2_norm	pR2/max(pR2)
R2_norm	R2/max(R2)
HGF_norm	HGF/max(HGF)
cMet_norm	cMet ./ max (cMet)
mVEGFA_norm	mVEGFA./ max(mVEGFA)
pAkt_Akt_norm	(pAkt ./Akt) ./ max(pAkt ./ Akt)
pERK_ERK_norm	(pERK ./ ERK) ./ max (pERK ./ ERK)
peNOS_eNOS_nor m	(peNOS ./ eNOS) ./ max(peNOS ./ eNOS)
Ca_norm	c1.Ca/max(c1.Ca)
NO_norm	NO/max(NO)
pPLCy_norm	pPLCy/max(pPLCy)
peNOS_norm	peNOS/max(peNOS)
pAkt_norm	pAkt/max(pAkt)
pERK_norm	pERK/max(pERK)
pSrc_norm	pTSADSrc ./ max (pTSADSrc)
s6_s60	pS6 ./ pS6(1)
mTORC1_mTORC1_ 0	ac_mTORC1 ./ ac_mTORC1(1)
pAkt_pAkt_0	pAkt ./ pAkt(1)
pERK_pERK_0	pERK ./ pERK(1)
PbI	((NO./max(NO)) .* (pS6./max(pS6)))./ (((S1P./max(S1P)) .*(pERK./max(pERK)))+k.*k)
PrI	(((pERK./max(pERK)) .* (pS6./max(pS6))) ./ (PTEN./max(PTEN)))
pS6_norm	pS6 ./ max (pS6)
S1P_norm	S1P ./ max (S1P)
pAkt_Akt_t0	(pAkt./Akt)./(pAkt(1)./Akt(1))
pERK_ERK_t0	(pERK./ERK)./(pERK(1)./ERK(1))
PbI2	((NO .* (pS6)) ./ (((S1P) .*k)+(k.*k))) ./ max (((NO .* (pS6)) ./ (((S1P) .*k)+(k.*k))))
**PrI_nonNorm**	((pERK .* pS6)+k.*k4)./((PTEN.*k) +k.*k4)
PbI3	(((Pak_v_sc+k)) ./ (((Pak_h_sc +k))))
PbI4	((NO .* k.*PAK_vegf) +(k.*k.*k2))./ ((S1P.*PAK_hgf.*pERK)+(k.*k.*k2))
TER1	1./PbI3
TER2	1./PbI4

s1	PAK_vegf./PAK_vegf(1)
s2	PAK_hgf./PAK_hgf(1)
Pak_v	PAK_vegf./PAK_vegf(1)
Pak_h	PAK_hgf ./ PAK_hgf(1)
Pak_h_sc	PAK_hgf
Pak_v_sc	PAK_vegf
S1P_sc	S1P
**PbI5**	(((NO .*k) +(k.*k5))./ ((S1P.*pERK)+(k.*k5)))
PbI4norm	PbI4./max(PbI4)

Bold font represents Observables used on PbI and PrI calculations

Supplementary table S2. Table of structural identifiability analysis

Parameter	GenSSI result	STRIKE-GOLDD result
kp_R2	Unidentifiable	Identifiable
kdp_R2	Unidentifiable	Identifiable
kf_V_R2	Unidentifiable	Identifiable
kf_dim	Identifiable	Identifiable
kr_dim	Identifiable	Identifiable
kint_HIF	Identifiable	Identifiable
ktransl_mHIF1a	Identifiable	Identifiable
kdeg_HIF1a	Identifiable	Identifiable
kform_mVEGFA	Unidentifiable	Identifiable
kdeg_mVEGFA	Unidentifiable	Identifiable
kp_cMet	Unidentifiable	Identifiable
kf_pMetGab2	Unidentifiable	Identifiable
kf_pMetGab1	Unidentifiable	Identifiable
kr_pMetGab1	Unidentifiable	Identifiable
kr_pMetGab2	Unidentifiable	Identifiable
kf_Gab2SHP	Unidentifiable	Identifiable
kr_Gab2SHP	Unidentifiable	Identifiable
kf_Gab1SHP2	Unidentifiable	Identifiable
kr_Gab1SHP2	Unidentifiable	Identifiable
km_vegfcMet	Unidentifiable	Identifiable
km_hgfr2	Unidentifiable	Identifiable
Vm_mMet	Unidentifiable	Identifiable
km_hgfr2_met	Unidentifiable	Identifiable
kgen_R2	Identifiable	Identifiable
kdegR2	Identifiable	Identifiable

kf_eNOSCaM	Identifiable	Identifiable
Ipmca	Identifiable	Identifiable
I_ERCA	Identifiable	Identifiable
kf_CaNaEx	Identifiable	Identifiable
KmS1PRas	Unidentifiable	Unidentifiable
kcatERK	Identifiable	Identifiable
kf_actGabSOS	Unidentifiable	Identifiable
kdeg_eNOS	Identifiable	Identifiable
kf_paktHsp	Identifiable	Identifiable
konCaCIB1	Identifiable	Identifiable
KmMEKERK	Identifiable	Identifiable
kf_pAktmTORC1	Identifiable	Identifiable
kdeact_mTORC1	Identifiable	Identifiable
kf_mTORC1S6K1	Identifiable	Identifiable
kdeac_s6k1	Identifiable	Identifiable
kdeg_NO	Identifiable	Identifiable
kf_Gab1Plcy	Unidentifiable	Identifiable
kdeg_pGab1SHP2	Unidentifiable	Identifiable
kf_SrcPAK	Unidentifiable	Identifiable
kf_PI3KPAK	Unidentifiable	Identifiable
**Total**	21	44

Supplementary table S3. Table of practical identifiability analysis

Structurally Identifiable parameters	Collinearity	PIA- PRCC	Fix or fit
kdp_R2	Fixed	Fixed	Fixed
kr_dim	Fixed	Fixed	Fixed
kdeg_mVEGFA	Fixed	Fixed	Fixed
kr_pMetGab1	Fixed	Fixed	Fixed
kr_pMetGab2	Fixed	Fixed	Fixed
kr_Gab2SHP	Fixed	Fixed	Fixed
kr_Gab1SHP2	Fixed	Fixed	Fixed
km_vegfcMet	Fixed	Fixed	Fixed
km_hgfr2	Fixed	Fixed	Fixed
km_hgfr2_met	Fixed	Fixed	Fixed
kgen_R2	Fixed	Fixed	Fixed
I_ERCA	Fixed	Fixed	Fixed
kdeact_mTORC1	Fixed	Fixed	Fixed
kdeac_s6k1	Fixed	Fixed	Fixed
kp_R2	Evaluated	Influential	Fit

kf_CaNaEx	Evaluated	Influential	Fit
kcatERK	Evaluated	Influential	Fit
kf_dim	Evaluated	Influential	Fit
kf_actGabSOS	Evaluated	Low PRCC	Fixed
kdeg_eNOS	Evaluated	Low PRCC	Fixed
kf_paktHsp	Evaluated	Influential	Fit
konCaCIB1	Evaluated	Influential	Fit
KmMEKERK	Evaluated	Influential	Fit
kf_pAktmTORC1	Evaluated	Influential	Fit
kf_mTORC1S6K1	Evaluated	Low PRCC	Fixed
kdeg_NO	Evaluated	Influential	Fit
ktransl_mHIF1a	Evaluated	Influential	Fit
kdeg_HIF1a	Evaluated	Influential	Fit
kform_mVEGFA	Evaluated	Influential	Fit
kf_V_R2	Evaluated	Influential	Fit
kp_cMet	Evaluated	Influential	Fit
kf_pMetGab2	Evaluated	Influential	Fit
kf_pMetGab1	Evaluated	Influential	Fit
kf_Gab2SHP	Evaluated	Influential	Fit
kf_Gab1SHP2	Evaluated	Influential	Fit
Vm_mMet	Evaluated	Influential	Fit
kdegR2	Evaluated	Influential	Fit
kf_eNOSCaM	Evaluated	Influential	Fit
Ipmca	Evaluated	Influential	Fit
kint_HIF	Evaluated	Influential	Fit
kf_Gab1Plcy	Evaluated	Influential	Fit
kdeg_pGab1SHP2	Evaluated	Influential	Fit
kf_SrcPAK	Evaluated	Influential	Fit
kf_PI3KPAK	Evaluated	Low PRCC	Fixed

Supplementary table S4. Table of doses

Name	Target Name	Start Time	Amount	Amount Units	Time Units
V80 dose	c1.V	0	0.002	micromole	hour
V50 dose	c1.V	0	0.0012	micromole	hour
H84 dose	c1.HGF	0	0.001	micromole	hour
H0.84 dose	c1.HGF	0	0.00001	micromole	hour
H20 dose	c1.HGF	0	0.000238	micromole	hour
H10 dose	c1.HGF	0	0.00012	micromole	hour
V10 dose	c1.V	0	0.00024	micromole	hour

V20 dose	c1.V	0	0.00048	micromole	hour
V2.5 dose	c1.V	0	0.000059	micromole	hour
H40 dose	c1.HGF	0	0.00048	micromole	hour
V25 dose	c1.V	0	0.0006	micromole	hour
H25 dose	c1.HGF	0	0.000297	micromole	hour
H50 dose	c1.HGF	0	0.000595	micromole	hour
dose V	c1.V	0	0.0012	micromole	hour
dose H	c1.HGF	0	0.000595	micromole	hour
H1 dose	c1.HGF	0	1.19E-05	micromole	hour
Note:					
* Doses set as active depending on the fitting or simulation performed					
	VEGFa 165	HGF			
Molecular Weight	42kDa	84kDa			

Supplementary table S5. Table of rules

Name	Rule	Rule Type
Extracellular Ca flux	jcrac = (((Caext*Volext/Volcyto-c1.Ca)*((Icrac_2*Kcrac_2^4/(Kcrac_2^4+c1.Ca_ER^4))))-jcrac)/tau_stim2	rate

Supplementary table S6. Table of species

Name	Value	Units	Constant
PHD2_protein	0.009	micromole	FALSE
PHD3_protein	0.009	micromole	FALSE
HIF1A_mrna	0.1	micromole	FALSE
HIF2A_mrna	0.5	micromole	FALSE
HIF1A_protein	0	micromole	FALSE
HIF2A_protein	0	micromole	FALSE
HIF1a_n	0	micromole	FALSE
HIF2a_n	0	micromole	FALSE
HIF1b_n	0.1	micromole	TRUE
HIF1ab	0	micromole	FALSE
HIF2ab	0	micromole	FALSE
mVEGFA	0	micromole	FALSE

mR2	0.001	micromole	FALSE
R2	0.0099	micromole	FALSE
V	0	micromole	FALSE
VR2	0	micromole	FALSE
pR2	0	micromole	FALSE
iR2	0	micromole	FALSE
iVR2	0	micromole	FALSE
HGF	0	micromole	FALSE
cMet	0.0166	micromole	FALSE
HGF_cMet	0	micromole	FALSE
p_cMet	0	micromole	FALSE
Gab1	0.17	micromole	FALSE
Gab2	0.17	micromole	FALSE
pGab2	0	micromole	FALSE
pGab1	0	micromole	FALSE
Grb2	0.0021	micromole	FALSE
stableActGab1	0	micromole	FALSE
SHP2	1.7	micromole	FALSE
Gab2SHP2	0	micromole	FALSE
p_Gab1SHP2	0	micromole	FALSE
mcMet	0.001	micromole	FALSE
PIP2	10	micromole	FALSE
PI3kin	0.1	micromole	FALSE
PI3Kac	0	micromole	FALSE
PIP3	0	micromole	FALSE
PTEN	0.01	micromole	FALSE
PDK1_PIP3	0	micromole	FALSE
ppAkt	0	micromole	FALSE
pAkt	5E-08	micromole	FALSE
mTORC2_ac	100	micromole	FALSE
Akt	1	micromole	FALSE
Akt_PIP3	0	micromole	FALSE
TsadSrc	0.1	micromole	FALSE
pTSADSrc	0	micromole	FALSE
Axl	10	micromole	FALSE
pAxl	0	micromole	FALSE
ppAxl	0	micromole	FALSE
ac_mTORC1	5E-05	micromole	FALSE
pS6	0.0001	micromole	FALSE
PLCy	0.2	micromole	FALSE
pPLCy	0	micromole	FALSE

IP3	0	micromole	FALSE
DAG	0	micromole	FALSE
eNOS	0.1	micromole	FALSE
CaM	1	micromole	FALSE
Ca	0.05	micromole	FALSE
CaCaM	0	micromole	FALSE
NO	0.0005	micromole	FALSE
Arg	100	micromole	FALSE
eNOS_CaM	0	micromole	FALSE
CaF	118.03	micromole	FALSE
CaCaF	1.97	micromole	FALSE
Ca_ER	2000	micromole	FALSE
PKC	0.1	micromole	FALSE
PKC_Ca	0	micromole	FALSE
Cit	0	micromole	FALSE
PKC_DAG	0	micromole	FALSE
HSP90	0.5	micromole	FALSE
acHSP90	0	micromole	FALSE
HSP_CaMeNOS	0	micromole	FALSE
pERK	3E-05	micromole	FALSE
MEK12	0.289	micromole	FALSE
ppMEK12	0	micromole	FALSE
ERK	0.382	micromole	FALSE
SphK	0.1	micromole	FALSE
CIB1Ca	0	micromole	FALSE
CIB1	0.5	micromole	FALSE
pSphK	0	micromole	FALSE
CIB1_SphK	0	micromole	FALSE
CIB1SphK_memb	0	micromole	FALSE
Sph	10	micromole	FALSE
S1P	1E-10	micromole	FALSE
RafRasGTP	0	micromole	FALSE
RasGTP	0	micromole	FALSE
Raf	0.355	micromole	FALSE
ppRaf	0	micromole	FALSE
PDK1	0.1	micromole	FALSE
peNOS	0	micromole	FALSE
PAK_vegf	1E-08	micromole	FALSE
PAK_hgf	1E-10	micromole	FALSE
PAK	0.01	micromole	FALSE

Supplementary table S7. Table of variants

Type	Name	hpx
parameter	O2	9.9

Supplementary table S8. ELISA protocol

Name	Detail
Software Version	3.10.06
Experiment File Path:	C:\Users\RTCAdmin\Desktop\Plate Reader Data\Akash\230526 HUVEC Hypoxia HGF ELISA\230526 HUVEC Hypoxia HGF ELISA.xpt
Protocol File Path:	C:\Users\Public\Documents\Protocols\ELISA_IL8.prt
Plate Number	Plate 1
Date	5/26/2023
Time	17:01:16
Reader Type:	Synergy H1
Reader Serial Number:	1611028
Reading Type	Reader
**Procedure Details**	
Plate Type	96 WELL PLATE
Eject plate on completion	
Read	Absorbance Endpoint
	Full Plate
	Wavelengths: 450, 540
	Read Speed: Normal, Delay: 100 msec, Measurements/Data Point: 8
**Results**	
Actual Temperature:	24.7
Actual Temperature:	24.7

	1	2	3	4	5	6	7	8	9	10	11	12	
A	2.233	2.14	2.487	1.387	2.03	0.95	0	0	0	0	0	0.008	450
	0.082	0.073	0.106	0.078	0.133	0.064	0	0	0	0	0	0.007	540
B	1.02	1.179	1.05	0.132	0.195	0.496	0	0	0	0	0	0	450
	0.059	0.06	0.063	0.044	0.043	0.052	0	0	0	0	0	0	540
C	0.617	0.555	0.561	0.144	0.163	0.238	0	0	0	0	0	0	450
	0.051	0.056	0.055	0.046	0.074	0.052	0	0	0	0	0	0	540
D	0.357	0.393	0.34	0.141	0.197	0.257	0	0	0	0	0	0	450
	0.057	0.051	0.049	0.044	0.057	0.05	0	0	0	0	0	0	540
E	0.243	0.247	0.215	0.124	0.12	0.167	0	0	0	0	0	0	450

	0.066	0.048	0.046	0.046	0.036	0.053	0	0	0	0	0	0	540
F	0.184	0.195	0.168	0.115	0.117	0.187	0	0	0	0	0	0	450
	0.05	0.046	0.045	0.037	0.037	0.051	0	0	0	0	0	0	540
G	0.118	0.118	0.107	0.122	0.108	0.218	0	0	0	0	0	0	450
	0.051	0.049	0.039	0.034	0.035	0.047	0	0	0	0	0	0	540
H	0.074	0.079	0.093	0.111	0.167	0.138	0	0	0	0	0	0	450
	0.043	0.046	0.045	0.033	0.035	0.046	0	0	0	0	0	0	540

Supplementary table S9. ELISA analysis

	Standards			Samples			Hypoxia Duration (h)
10 µg/mL	2.233	2.14	2.487	1.387	2.03	0.95	0 h
	0.082	0.073	0.106	0.078	0.133	0.064	
5 µg/mL	1.02	1.179	1.05	0.132	0.195	0.496	1 h
	0.059	0.06	0.063	0.044	0.043	0.052	
2.5 µg/mL	0.617	0.555	0.561	0.144	0.163	0.238	2 h
	0.051	0.056	0.055	0.046	0.074	0.052	
1.25 µg/mL	0.357	0.393	0.34	0.141	0.197	0.257	4 h
	0.057	0.051	0.049	0.044	0.057	0.05	
625 pg/mL	0.243	0.247	0.215	0.124	0.12	0.167	6 h
	0.066	0.048	0.046	0.046	0.036	0.053	
313 pg/mL	0.184	0.195	0.168	0.115	0.117	0.187	8 h
	0.05	0.046	0.045	0.037	0.037	0.051	
156 pg/mL	0.118	0.118	0.107	0.122	0.108	0.218	24 h
	0.051	0.049	0.039	0.034	0.035	0.047	
0 pg/mL	0.074	0.079	0.093				
	0.043	0.046	0.045				
	A450 - A540						
10 µg/mL	2.151	2.067	2.381	1.309	1.897	0.886	
5 µg/mL	0.961	1.119	0.987	0.088	0.152	0.444	
2.5 µg/mL	0.566	0.499	0.506	0.098	0.089	0.186	

1.25 µg/mL	0.3	0.342	0.291	0.097	0.14	0.207	
625 pg/mL	0.177	0.199	0.169	0.078	0.084	0.114	
313 pg/mL	0.134	0.149	0.123	0.078	0.08	0.136	
156 pg/mL	0.067	0.069	0.068	0.088	0.073	0.171	
0 pg/mL	0.031	0.033	0.048				
Conc (pg/mL)	Average O.D.		Hypoxia Duration (h)	Average O.D.	SD	**HGF Concentration (pg/mL)**	**SD**
10000	2.199667		0	1.364	0.414567	**6266.558**	**1951.904885**
5000	1.022333		1	0.228	0.154954	**917.9346**	**729.5679979**
2500	0.523667		2	0.124333	0.043759	**429.8413**	**206.0323689**
1250	0.311		4	0.148	0.045262	**541.271**	**213.1077859**
625	0.181667		6	0.092	0.015748	**277.6065**	**74.14630388**
313	0.135333		8	0.098	0.026882	**305.8562**	**126.5705531**
156	0.068		24	0.110667	0.043099	**365.4946**	**202.9245409**
0	0.037333						
Lin. Reg.	0.000212	0.033039					
